# TSP50 attenuates metabolic dysfunction-associated steatotic liver disease via SCD1 degradation-mediated suppression of hepatocyte lipogenesis

**DOI:** 10.1186/s11658-026-00859-2

**Published:** 2026-02-01

**Authors:** Jiujia Liang, Zhihui Luan, Rong Jin, Rina Su, Jiarong Ge, Xiao Tian, Chunxue Niu, Jiawei Li, Xiaoli Li, Feng Gao, Zhenbo Song, Luguo Sun, Guannan Wang, Lihua Zheng, Ying Sun, Lei Liu, Yongli Bao, Shuyue Wang, Xiaoguang Yang

**Affiliations:** 1 National Engineering Laboratory for Druggable Gene and Protein Screening, Northeast Normal University, Changchun, 130117 China; 2 China International Joint Research Center for Human Stem Cell Bank, Northeast Normal University, Changchun, 130024 China; 3 Key Laboratory of Molecular Epigenetics, Institute of Genetics and Cytology, Ministry of Education, Northeast Normal University, Changchun, 130024, China; 4 School of Life Sciences, Changchun Normal University, Changchun, 130032 China

**Keywords:** TSP50, SCD1, Protease, MASLD, Hepatocyte lipid accumulation

## Abstract

**Background:**

Metabolic dysfunction-associated steatotic liver disease (MASLD) is a major contributor to chronic liver disease worldwide, yet the molecular mechanisms driving its pathogenesis remain incompletely defined. Although dysregulated hepatic lipogenesis is a well-established driver of MASLD progression, the role of testes-specific protease 50 (TSP50)—an enzyme with demonstrated oncogenic functions in multiple cancers—in hepatic lipid metabolism and its potential involvement in the development of MASLD remains unexplored.

**Methods:**

The study utilized the STelic Animal Model (STAM) along with high-fat/high-cholesterol plus fructose (HFF) and methionine-choline deficient (HFMCD) dietary models to evaluate the functional role of TSP50 in MASLD progression. Hepatocyte-specific knockout and AAV-mediated TSP50 reconstitution were performed to assess cell-autonomous effects. Mechanistic insights were gained through biochemical analyses of lipid metabolism pathways and protein interaction studies.

**Results:**

TSP50 deficiency markedly accelerated MASLD progression across all experimental models, promoting hepatic steatosis, inflammation and fibrosis while increasing susceptibility to hepatocellular carcinoma (HCC). Conversely, TSP50 supplementation exerted protective effects against MASLD development. Furthermore, we identified a novel regulatory mechanism whereby TSP50 directly interacts with and degrades stearoyl-CoA desaturase 1 (SCD1) through its catalytic hydrolase activity, thereby suppressing de novo lipogenesis. The inhibitor of SCD1 rescued hepatic TSP50 knockout induced lipid accumulation and liver injury during MASLD.

**Conclusions:**

Our study reveals the role of TSP50 in hepatic lipid metabolism, identifying it as a novel regulator of hepatic de novo lipogenesis that exerts protective effects against MASLD through catalytic degradation of SCD1. These findings not only advance our understanding of MASLD pathogenesis but also offer novel insights for developing therapeutic strategies.

**Supplementary Information:**

The online version contains supplementary material available at 10.1186/s11658-026-00859-2.

## Background

Nonalcoholic fatty liver disease (NAFLD), now referred to as metabolic dysfunction-associated steatotic liver disease (MASLD) has emerged as a global health crisis, affecting approximately 25% of the population worldwide [1]. MASLD begins with excessive lipid accumulation in hepatocytes, and 20% to 30% of individuals with MASLD will develop metabolic dysfunction-associated steatohepatitis (MASH), formerly known as nonalcoholic steatohepatitis (NASH), which is characterized by liver injury, inflammation, and fibrosis [2]. If left untreated, MASH can lead to the development of cirrhosis and hepatocellular carcinoma (HCC) [3, 4]. Despite its high prevalence, therapeutic options remain limited. The US Food and Drug Administration (FDA) recently approved Resmetirom, a selective agonist targeting the thyroid hormone receptor β, as the first medication for MASH with fibrosis. However, only roughly 25% of patients experience benefits from this therapy [5, 6]. The mechanisms underlying MASLD remain incompletely understood, and there is a pressing need for identifying effective therapeutic targets to advance clinical drug development [7–9]. Intracellular accumulation of lipids, especially triglycerides is the fundamental feature of MASLD [10].

The imbalance between fatty acid synthesis and degradation is a key contributor to hepatic lipid accumulation [11, 12]. Metabolic enzymes tightly regulate the processes of fatty acid synthesis and catabolism. In the liver tissue of patients with MASH, elevated expression levels of key enzymes involved in de novo lipogenesis (DNL) are frequently detected [13]. Stearoyl-CoA desaturase 1 (SCD1) is an essential enzyme in DNL, as it catalyzes the conversion of saturated fatty acids (SFAs) to monounsaturated fatty acids (MUFAs) [14, 15]. The MUFA (C18:1) can upregulate the mRNA levels of sterol regulatory element-binding protein 1 (SREBP1), fatty acid synthase (FASN), and acetyl-CoA carboxylase (ACC) [16]. The inhibition of liver SCD1, either through the use of oral inhibitors or small interfering RNA (siRNA), has been shown to effectively reduce steatosis and fibrosis in animal models of MASLD [17–19]. Multiple SCD1 inhibitors have been developed for MASH treatment and have shown significant therapeutic benefits [20, 21].

Intriguingly, testes-specific protease 50 (TSP50), as a threonine protease, has recently garnered attention for its role in metabolic regulation [22–26]. Under physiological conditions, TSP50 is predominantly expressed in the testis, thyroid, and pituitary gland, with lower levels observed in the liver and other tissues [27]. However, aberrant overexpression has been detected in various cancers, including hepatocellular carcinoma [25, 28–30]. Previous studies have demonstrated that TSP50 regulates tumor proliferation through its protease activity [31]. However, its role in MASLD pathogenesis—particularly in lipid metabolism—remains unexplored. This study aims to investigate the role of TSP50 in MASLD progression and to delineate the mechanistic link between TSP50 and SCD1 in hepatocyte lipid accumulation, potentially identifying novel therapeutic targets for MASLD.

## Materials and methods

### Animals and genotyping

All experimental procedures were reviewed and approved by the Animal Advisory Committee at Northeast Normal University, China. Mice were housed under specific pathogen-free (SPF) conditions. All of the mice were maintained under a 12 h light/12 h dark cycle. The mice were mated, bred, and genotyped in the Animal Experimental Center of National Engineering Laboratory for Druggable Gene and Protein Screening. All of the mice used in this study were maintained on a C57BL/6N genetic background.

*Tsp50*^−/−^ mice and *Tsp50*^*fl/fl*^ mice were generated as previously described [32, 33].

*Tsp50*^*fl/fl*^*Alb*^*cre*^ mice: *Tsp50*^*fl/fl*^ mice and AlbCreERT2 mice were purchased from GemPharmatech Co., Ltd (Nanjing, China). Heterozygous animals carrying one floxed (fl; flanked by loxP) and one wild-type allele were crossed with animal hemizygous albumin-Cre transgene (AlbCreERT2) purchased from GemPharmatech Co. Ltd, (Nanjing, China). Heterozygous (fl/+) animals carrying one copy of the AlbCreERT2 transgene were then interbred with fl/+ littermates lacking Cre to generate TSP50 hepatocyte-specific knockout mice and littermate control mice. Knockout of TSP50 was induced by intraperitoneal injection of tamoxifen (80 mg/kg/per day) for 5 days starting 2 weeks before the start of the experiment.

### MASLD models, AAV9 injection, and pharmacological treatment

#### STAM-MASLD

STAM mice was generated by subcutaneous injection of low-dose streptozotocin (STZ, 200 μg per mouse, Beijing solarbio Co. Ltd, China) to 2-day-old male mice. This was followed by feeding them a High Fat Diet 32 (consisting of 25.5% protein, 32.0% fat, 29.4% nitrogen-free extract, 4.0% ash, 2.9% fiber, and 6.2% water, HFD32, Jiangsu medicine Co. Ltd, China) starting from the age of 4 weeks.

#### HFF-MASH

HFF-MASH mouse model was established by feeding 6–8 weeks old male mice high-fat/high-cholesterol plus high fructose (HFF) diet (20 kcal% protein, 40 kcal% fat, 20 kcal% fructose and 2% cholesterol, Jiangsu medicine Co. Ltd, China) for 24 weeks.

#### HFMCD-MASH

HFMCD-MASH mouse model was established by feeding 6–8 weeks old male mice high-fat with methionine and choline deficiency (HFMCD) diet (60 kcal% fat with low methionine and no choline, Jiangsu medicine Co. Ltd, China) for 6 weeks.

#### AAV9 injection

For AAV9-mediated gene delivery, a tail vein injection was performed in 4 week-old male mice using AAV-mCherry or AAV-mCherry-TSP50-Flag vectors (1 × 10^11^ genome copies per mouse, provided by Shanghai Just science Co. Ltd, China). After 2 weeks, the mice were subjected to 24 weeks of HFF feeding.

#### Pharmacological treatment

For administration of the SCD1 inhibitor, 10 week-old male mice were given MK-8245 via gavage at a dose of 10 mg/kg/, once weekly for a period of 4 weeks.

All experiments described above were performed using male mice.

### Cell culture and plasmid transfection

HepG2 (Cat.No. GNHu17) cells and HEK-293 T (Cat.No. SCSP-510) cells were obtained from the cell library of the National Collection of Authenticated Cell Cultures (Shanghai, China). THLE2 (Cat.No. CTCC-004–0030) cells were obtained from the Zhejiang MEISEN Cell Technology Co. Ltd, (Zhejiang, China) HepG2 and HEK-293 T cells were cultured inHigh-glucose Dulbecco’s Modified Eagle Medium(H-DMEM) supplemented with 10% fetal bovine serum (FBS). THLE2 cells were cultured based in BEGM™ BulletKit™ medium (Lonza, CC-3170). The kit includes 500 mL basal medium and separate frozen additives from which we discard the gentamycin/amphotericin (GA) and epinephrine and to which we added extra 5 ng/mL EGF, 70 ng/mL phosphoethanolamine, and 10% fetal bovine serum. All cell lines were cultured at 37 °C with 5% CO_2_.

Upon reaching 80% confluence, a transfection procedure was conducted. Specifically, 200 µL of OPTI-MEM medium was mixed with 5 µL of lipofectamine 2000 and 2.5 µg of plasmid DNA. After incubating the transfection mixture at room temperature for 30 min, it was added to each well containing the cultured cells. Following a 5-h incubation period, the medium was replaced with the corresponding complete medium.

### Biochemical assay

Triglycerides (TG) and total cholesterol (TCHO) in mouse liver, serum, or cells were measured using commercial kits (Nanjing Jiancheng Co. Ltd, China). Alanine aminotransferase (ALT) and aspartate aminotransferase (AST) levels in mice serum were measured using commercial kits (Nanjing Jiancheng Co. Ltd, China).

### Histological analyses

Liver sections were embedded in paraffin and then stained with hematoxylin and eosin (H&E) to visualize the pattern of lipid accumulation and inflammatory state. The extent of fibrosis was assessed using Sirius Red staining. Lipid droplet accumulation was visualized using Oil Red O staining of frozen liver sections prepared in Tissue-Tek OCT compounds. The positive staining area in each picture was quantified by ImageJ Pro Plus software.

### Western blot analysis

Cell and tissue lysates were subjected to Sodium Dodecyl Sulfate–Polyacrylamide Gel Electrophoresis (SDS-PAGE) electrophoresis, transferred to nitrocellulose or polyvinylidene difluoride (PVDF) membranes, and then blotted with antibodies. Chemiluminescent color development of the immunoblots was performed using the Enhanced Chemiluminescence Western Blotting System. Band intensity was measured for quantification using ImageJ software.

### Immunohistochemistry

Paraffin-embedded mouse liver sections were dewaxed and hydrated, and high-pressure antigen repair was performed using sodium citrate antigen repair solution or Tris–EDTA antigen repair solution (Beijing solarbio Co. Ltd, China). The reaction was performed using the corresponding primary antibody as well as the corresponding secondary antibody detection kit (Beijing ZSGB-bio–Co. Ltd, China), and then the DAB color development kit was used for color development (Beijing ZSGB-bio–Co. Ltd, China). The slides were stained with hematoxylin, dehydrated, and mounted for bright-field microscopy. The F4/80 positive area was analyzed using ImageJ software.

### Immunofluorescence

The mouse liver tissues were fixed in 4% paraformaldehyde for 12 h at 4 °C. Following fixation, the tissues were soaked in phosphate buffered saline (PBS) containing 30% sucrose overnight at 4 °C. Subsequently, the tissues were embedded in optimal cutting temperature compound (OCT), frozen, and cut into 5 µm sections. The sections were blocked with 2% bovine serum albumin (BSA) in 0.2% Triton X-100/PBS for 1 h and then incubated with primary antibodies overnight at 4 °C. Following five washes with PBS, the sections were incubated with appropriate fluorochrome-conjugated secondary antibodies for 1 h, followed by another five washes. Coverslips were mounted onto glass slides using 75% glycerol as a mounting medium. Images were acquired using a laser scanning confocal microscope (Zeiss, Germany).

### Measurement of fatty acid composition by gas chromatography–mass spectrometry (GC–MS)

The samples were thawed at 4 ℃ and 50 mg of each sample was mixed with 5 mL of cold methylene dichloride-methanol (2:1 v/v) solution, adequately vortexed, then sonicated for 30 min at a low temperature, washed with 2 mL gold standard water, and the lower solution was taken and dried with nitrogen. The mixture was mixed with internal standard and 2 mL n-hexane to achieve fatty-acid esterification for methyl esterification for 30 min. Then, 2 mL gold standard water was added and adequately vortex. The supernatant (1000 µL) was dried with nitrogen, redissolved in n-hexane. The supernatants were collected for GC–MS analysis.

The samples were separated with an Agilent DB-23 GC column (60 m × 250 μm × 0.15 μm). The initial temperature was 80 °C, then increased to 180 °C at 20 °C/min, and remained at 220 °C for 8 min. Then the temperature was increased to 280 ℃ at 5 ℃/min, and remained as such for 3 min. The carrier gas was helium, and the carrier gas flow rate was 1.0 mL/min. The quality control (QC) samples were used for testing and evaluating the stability and repeatability of this system. A5977BMSD mass spectrometer (Agilent) was used for mass spectrum analysis. 5977BMSD conditions are as follows: inlet temperature: 280 ℃; ion source temperature: 230 ℃.

### Coimmunoprecipitation (Co-IP) analysis

For Co-IP assays, cultured HepG2 cells and THLE2 cells were cotransfected with the specified plasmids for 36 h and then lysed in 1 mL of lysis buffer for Western and IP (P0013, Shanghai Beyotime Biotechnology Co. Ltd, China). Each sample consisting of 400 µL of lysate was incubated with 2 mg of the specified antibody overnight at 4 °C with rocking, followed by incubation with 50 μL of Protein A/G Magnetic Beads (HY-K0202, Shanghai MedChemExpress Co. Ltd, China) for 4 h. Finally, the beads were washed 5–6 times with cold IP buffer and antigen/antibody beads were resuspended in 60 µL of SDS loading buffer and subjected to western blot analysis.

### GST pull-down assay

The BL21 bacteria harboring the recombinant pGEX-4 T-1-GST-TSP50 plasmid were streaked onto the culture medium and incubated overnight at 37 °C. The following day, single colonies were selected and incubated overnight. Subsequently, these cultures were inoculated into fresh medium at an inoculum ratio of 1% and incubated at 37 °C with a shaking speed of 200 rpm until the optical density at 600 nm (OD600) reached a range of 0.6–1.0. Isopropyl-β-D-thiogalactopyranoside (IPTG) was then added to the medium at a final concentration of 1 mM to induce protein expression. After 5 h of induction, the bacteria were harvested by centrifugation. Finally, the GST-TSP50 protein was purified using the GST-tagged Protein Purification Kit (P2262, Shanghai Beyotime Biotechnology Co. Ltd, China) according to the manufacturer’s instructions.

For GST pull-down assays, cultured HEK293T cells were transfected with the specified plasmids for 36 h and then lysed in 1 mL of lysis buffer for Western and IP (P0013, Shanghai Beyotime Biotechnology Co. Ltd, China). Each sample consisting of 400 µL of lysate was incubated with GST-TSP50 protein overnight at 4 °C with rocking, followed by incubation with 50 μL of GST Magnetic Beads (HY-K0222, Shanghai MedChemExpress Co. Ltd, China) for 4 h. Finally, the antigen/antibody-bead complexes were resuspended in 60 µL of SDS loading buffer and subjected to western blot analysis.

### Correlation analysis of hepatic protein expression in patients with MASH

The datasets used in this study are sourced from the Gene Expression Omnibus (GEO) database (https://www.ncbi.nlm.nih.gov/geo/), with the downloaded data in MINiML format. The detailed processing procedure can be found in the method description on the dataset selection page. We utilized the ggstatsplot package in R software to generate the correlation plot for two genes, while the correlation plot for multiple genes was displayed using the pheatmap package in R software. We employed Spearman’s correlation analysis to describe the correlation between quantitative variables that do not follow a normal distribution. A *p*-value less than 0.05 was considered statistically significant.

### Statistical analysis

All statistical analyses of the population studies were performed using Prism version 9.0 software. For the animal and in vitro studies, the data are presented as the mean ± standard error of the mean (SEM). For experiments with two groups, we have performed unpaired two-tailed Student’s *t* tests. For comparisons among three or more independent groups, we have performed one-way analysis of variANCE (ANOVA) followed by Tukey’s multiple comparisons test. For experiments with two independent variables, we have performed two-way ANOVA followed by Šídák’s multiple comparisons test. The asterisks in the figures indicate statistical significance as follows: ∗*p* < *0.05;* ∗∗*p* < *0.01,* ∗∗∗*p* < *0.001* and ∗∗∗∗*p* < *0.0001.*

## Results

### Systemic TSP50 deficiency exacerbates MASLD progression to HCC

To elucidate the role of TSP50 in the progression of MASLD to HCC, we utilized the STAM to compare disease progression between TSP50 knockout (*Tsp50*^*−/−*^) mice and wild-type (WT) mice. In STAM mice, MASLD development was age-dependent, with both body weight and liver weight gradually increasing during the progression from MASLD to HCC. However, no significant differences were observed in the liver-to-body-weight ratio between *Tsp50*^*−/−*^ and WT mice (Supplementary Fig. S1A).

At 16 weeks of age, tumor nodules became visible on the liver surface of STAM mice. Notably, *Tsp50*^*−/−*^ mice exhibited a significantly higher number of tumor nodules compared with WT mice (Fig. S1B). Immunohistochemical (IHC) staining of liver tissue sections revealed positive staining for alpha-fetoprotein (AFP) and Ki67 in the tumor nodule areas, confirming the presence of HCC (Fig. S1C).

**Fig. 1:**
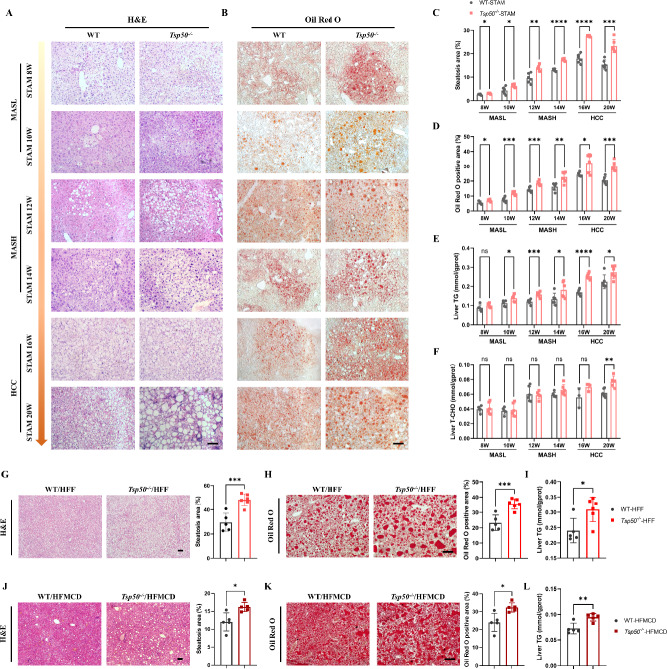
Systemic TSP50 deficiency exacerbates metabolic dysfunction-associated steatotic liver disease (MASLD) progression to hepatocellular carcinoma (HCC). **A** Hematoxylin and eosin (H&E) staining of liver sections from STAM-WT and STAM-*Tsp50*^*−/−*^ mice (*n* = 6 mice/group). Scale bars, 20 μm. **B** Oil Red O staining of liver sections from STAM-WT and STAM-*Tsp50*^*−/−*^ mice (*n* = 6 mice/group). Scale bars, 20 μm. **C** Quantification of steatosis in liver sections from STAM-WT and STAM -*Tsp50*^*−/−*^ mice (*n* = 6 mice/group). **D** Quantification of Oil Red O positive area quantification in liver sections from STAM-WT and STAM-*Tsp50*^*−/−*^ mice (*n* = 6 mice/group). **E** Hepatic triglyceride (TG) content of STAM-WT and STAM-*Tsp50*^*−/−*^ mice (*n* = 6 mice/group). **F** Hepatic total cholesterol (TCHO) content of STAM-WT and STAM-*Tsp50*^*−/−*^ mice (*n* = 6 mice/group). **G** H&E staining (left) and steatosis quantification (right) in liver sections from HFF/MASH-WT and HFF/MASH-*Tsp50*^*−/−*^ mice (*n* = 5 mice/group). Scale bars, 20 μm. **H** Oil Red O staining (left) and quantification (right) in liver sections from HFF/MASH-WT and HFF/MASH-*Tsp50*^*−/−*^ mice (*n* = 5 mice/group). Scale bars, 20 μm. **I** Hepatic TG content of HFF/MASH-WT and HFF/MASH-*Tsp50*^*−/−*^ mice (*n* = 5 mice/group). **J** H&E staining (left) and steatosis quantification (right) in liver sections from HFMCD/MASH-WT and HFMCD/MASH-*Tsp50*^*−/−*^ mice (*n* = 5 mice/group). Scale bars, 20 μm. **K** Oil Red O staining (left) and quantification (right) in liver sections from HFMCD/MASH-WT and HFMCD/MASH-*Tsp50*^*−/−*^ mice (*n* = 5 mice/group). Scale bars, 20 μm. **L** Hepatic TG content of HFMCD/MASH-WT and HFMCD/MASH-*Tsp50*^*−/−*^ mice (*n* = 5 mice/group)

To pinpoint the stage at which MASLD progression was accelerated by TSP50 knockout, we analyzed pathological staining results in STAM mice. *Tsp50*^*−/−*^ mice showed enhanced lipid deposition, as demonstrated by H&E and Oil Red O staining from the initial MASLD stage. (Figs. 1A–D). Subsequently, markedly exacerbated hepatic inflammation and fibrosis in *Tsp50*^*−/−*^ mice emerged during the MASH stage and progressively worsened throughout HCC development ( Figs. S1D–S1G).

We further examined the triglyceride (TG) and total cholesterol (TCHO) content in the liver tissues of STAM mice. TSP50 knockout consistently promoted TG accumulation in liver tissues throughout MASLD progression (Fig. 1E). The differences in TCHO content were only detected when MASLD progressed to HCC (Fig. 1F). Serum analysis revealed that TSP50 knockout elevated serum TG levels at the MASH stage; however, no significant differences in serum TCHO levels were observed between *Tsp50*^*−/−*^ and WT mice throughout MASLD development (Supplementary Figs. S1H, S1I). In addition, serum levels of liver injury biomarkers, such as alanine aminotransferase (ALT) and aspartate aminotransferase (AST), were significantly higher in *Tsp50*^*−/−*^ mice compared with WT mice at the MASH stage (Supplementary Fig. S1J). These findings collectively indicate that systemic TSP50 deficiency accelerates lipid accumulation, steatosis, inflammation, and fibrosis in the STAM model, exhibiting a stage-dependent phenotype that lipid accumulation initiates at the early MASLD stage, followed by progressive hepatic inflammation and fibrosis development upon MASH transition in *Tsp50*^*−/−*^ mice.

To further investigate the pathogenic role of TSP50 in MASH, we established diet-induced MASH models using two distinct nutritional regimens that recapitulate key features of human MASH progression: a high-fat/high-cholesterol plus high fructose diet (HFF) and a high-fat diet with methionine and choline deficiency (HFMCD). The results indicated that TSP50 knockout in mice led to increased lipid accumulation and steatosis under both dietary conditions (Figs. 1G–L). Under HFF diet-induced conditions, *Tsp50*^*−/−*^ mice exhibited significantly higher body weight and liver-to-body weight ratios compared with WT mice (Figs. S1K). Moreover, *Tsp50*^*−/−*^ mice showed more severe liver inflammation, fibrosis, and liver injury, as evidenced by histological and biochemical analyses (Supplementary Figs. S1L-S1P). The performance of *Tsp50*^*−/−*^ mice in HFMCD diet-induced MASH was consistent with that observed in the HFF diet model, with similarly exacerbated disease progression (Supplementary Figs. S1Q-S1S).

To determine whether TSP50 knockout promotes MASLD progression under normal physiological conditions, we examined the levels of lipid accumulation, steatosis, hepatic injury, and fibrosis in adult *Tsp50*^*−/−*^ mice reared under chow diet (Supplementary Figs. S1T-S1Y). Under these conditions, no significant differences were observed, suggesting that the promoting effect of TSP50 knockout on MASLD progression is primarily evident under metabolic stress conditions.

Collectively, these findings demonstrate that TSP50 knockout significantly accelerates MASLD/MASH progression to HCC. Moreover, TSP50 knockout markedly exacerbates hepatic steatosis in different MASH models, highlighting the critical role of TSP50 in modulating hepatic lipid metabolism.

### TSP50 deficiency in hepatocyte accelerates MASLD/MASH progression

**Fig. 2:**
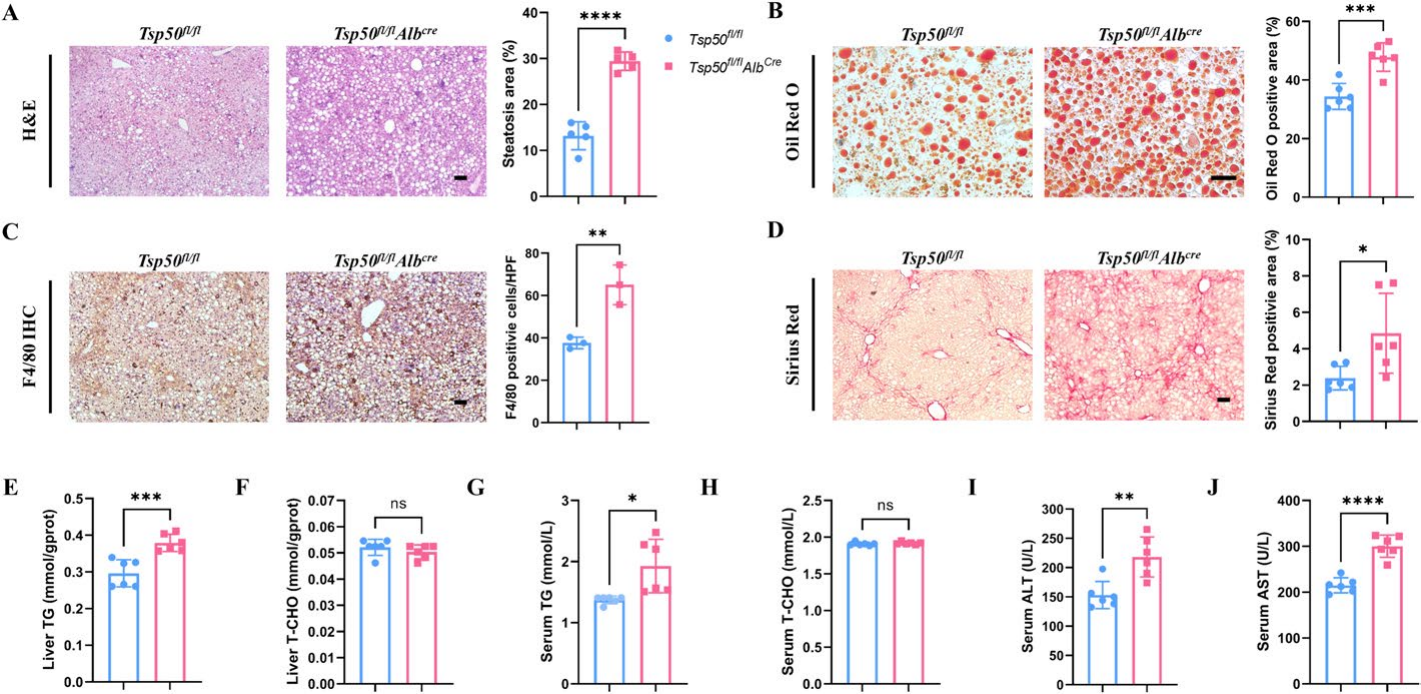
TSP50 deficiency in hepatocyte accelerates MASLD/MASH progression. **A** H&E staining (left) and steatosis quantification (right) in liver sections from HFMCD/MASH-*Tsp50*^*fl/fl*^ and HFMCD/MASH-*Tsp50*^*fl/fl*^*Alb*^*cre*^ mice (*n* = 5 mice/group). Scale bars, 20 μm. **B** Oil Red O staining (left) and quantification (right) in liver sections from HFMCD/MASH-*Tsp50*^*fl/fl*^ and HFMCD/MASH-*Tsp50*^*fl/fl*^*Alb*^*cre*^ mice (*n* = 5 mice/group). Scale bars, 20 μm. **C** F4/80 IHC positive area quantification in liver sections from HFMCD/MASH-*Tsp50*^*fl/fl*^ and HFMCD/MASH-*Tsp50*^*fl/fl*^*Alb*^*cre*^ mice (*n* = 3 mice/group). Scale bars, 20 μm. **D** Sirius Red staining (left) and quantification (right) in liver sections from HFMCD/MASH-*Tsp50*^*fl/fl*^ and HFMCD/MASH-*Tsp50*^*fl/fl*^*Alb*^*cre*^ mice (*n* = 5 mice/group). Scale bars, 20 μm. **E** Hepatic TG content of HFMCD/MASH-*Tsp50*^*fl/fl*^ and HFMCD/MASH-*Tsp50*^*fl/fl*^*Alb*^*cre*^ mice (*n* = 5 mice/group). **F** Hepatic TCHO content of HFMCD/MASH-*Tsp50*^*fl/fl*^ and HFMCD/MASH-*Tsp50*^*fl/fl*^*Alb*^*cre*^ mice (*n* = 5 mice/group). **G** Serum TG content of HFMCD/MASH-*Tsp50*^*fl/fl*^ and HFMCD/MASH-*Tsp50*^*fl/fl*^*Alb*^*cre*^ mice (*n* = 5 mice/group). **H** Serum TCHO content of HFMCD/MASH-*Tsp50*^*fl/fl*^ and HFMCD/MASH-*Tsp50*^*fl/fl*^*Alb*^*cre*^ mice (*n* = 5 mice/group). **I** Serum ALT level of HFMCD/MASH-*Tsp50*^*fl/fl*^ and HFMCD/MASH-*Tsp50*^*fl/fl*^*Alb*^*cre*^ mice (*n* = 5 mice/group). **J** Serum AST level of HFMCD/MASH-*Tsp50*^*fl/fl*^ and HFMCD/MASH-*Tsp50*^*fl/fl*^*Alb*^*cre*^ mice (*n* = 5 mice/group)

To further confirm the role of hepatocyte-specific TSP50 in the progression of MASLD to MASH, we generated hepatocyte-specific TSP50 knockout (*Tsp50*^*fl/fl*^*Alb*^*Cre*^) mice. These mice were subjected to HFMCD to induce MASH. Liver-to-body weight ratios did not differ significantly between *Tsp50*^*fl/fl*^ and *Tsp50*^*fl/fl*^*Alb*^*Cre*^ mice (Supplementary Fig. S2A). Notably, histological analyses revealed more severe hepatic lipid accumulation, steatosis, liver inflammation, and fibrosis in *Tsp50*^*fl/fl*^*Alb*^*Cre*^ mice compared with *Tsp50*^*fl/fl*^ mice (Figs. 2A–D). Biochemical analyses further supported these findings. The *Tsp50*^*fl/fl*^*Alb*^*Cre*^ mice exhibited significantly higher hepatic TG levels compared with *Tsp50*^*fl/fl*^ mice, while no significant differences were observed in hepatic TCHO and serum TCHO levels (Figs. 2E–H). Moreover, the serum levels of ALT and AST were significantly higher in *Tsp50*^*fl/fl*^*Alb*^*Cre*^ mice, indicating more severe liver injury after *Tsp50* deletion (Figs. 2I, J). Collectively, these findings highlight hepatocyte-specific TSP50 deficiency significantly accelerates the progression of MASLD/MASH, which is consistent with our observations in global TSP50 knockout mice.

### Complementation of TSP50 protects against MASLD/MASH progression

To further validate the role of TSP50 in the progression of MASLD to MASH, we employed an adeno-associated virus (AAV) type 9 vector to overexpress *Tsp50* in *Tsp50*^*−/−*^ mice. Specifically, AAV-mCherry and AAV-mCherry-TSP50-Flag (AAV-TSP50) were administered via tail vein injection at 4 weeks of age, followed by the introduction of a HFF diet at 6 weeks of age. Immunofluorescence analysis confirmed successful restoration of TSP50 expression in the livers of AAV-TSP50-treated mice (Supplementary Fig. S3A). Importantly, there was no significant difference detected in body weight and liver/body weight ratio between AAV-TSP50 mice and AAV-mCherry control mice (Supplementary Fig. S3B).

**Fig. 3:**
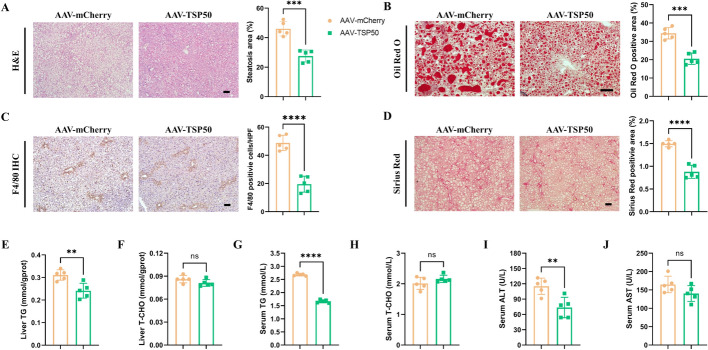
Complementation of TSP50 protects against MASLD/MASH progression. **A** H&E staining (left) and steatosis quantification (right) in liver sections from AAV-mCherry and AAV-TSP50 mice (*n* = 5 mice/group). Scale bars, 20 μm. **B** Oil Red O staining (left) and quantification (right) in liver sections from AAV-mCherry and AAV-TSP50 mice (*n* = 5 mice/group). Scale bars, 20 μm. **C** F4/80 IHC positive area quantification in liver sections from AAV-mCherry and AAV-TSP50 mice (*n* = 5 mice/group). Scale bars, 20 μm. **D** Sirius Red staining (left) and quantification (right) in liver sections from AAV-mCherry and AAV-TSP50 mice (*n* = 5 mice/group). Scale bars, 20 μm. **E** Hepatic TG content of AAV-mCherry and AAV-TSP50 mice (*n* = 5 mice/group). **F** Hepatic TCHO content of AAV-mCherry and AAV-TSP50 mice (*n* = 5 mice/group). **G** Serum TG content of AAV-mCherry and AAV-TSP50 mice (*n* = 5 mice/group). **H** Serum TCHO content of AAV-mCherry and AAV-TSP50 mice (*n* = 5 mice/group). **I** Serum ALT level of AAV-mCherry and AAV-TSP50 mice (*n* = 5 mice/group). **J** Serum AST level of AAV-mCherry and AAV-TSP50 mice (*n* = 5 mice/group)

Histological analyses revealed that AAV-TSP50 mice exhibited significantly reduced hepatic lipid accumulation, steatosis, liver inflammation and fibrosis compared with AAV-mCherry mice (Figs. 3A–D). The levels of hepatic TG and serum TG were significantly reduced in AAV-TSP50-treated mice, while no significant differences were observed in hepatic TCHO or serum TCHO levels (Figs. 3E–H). In addition, the serum levels of ALT and AST were significantly decreased in AAV-TSP*50* mice following HFF diet feeding (Figs. 3I, J). These findings indicate that TSP50 plays a regulatory role in lipid accumulation within hepatocytes during the progression of MASLD.

### TSP50 deficiency modifies the composition of hepatic fatty acids

**Fig. 4:**
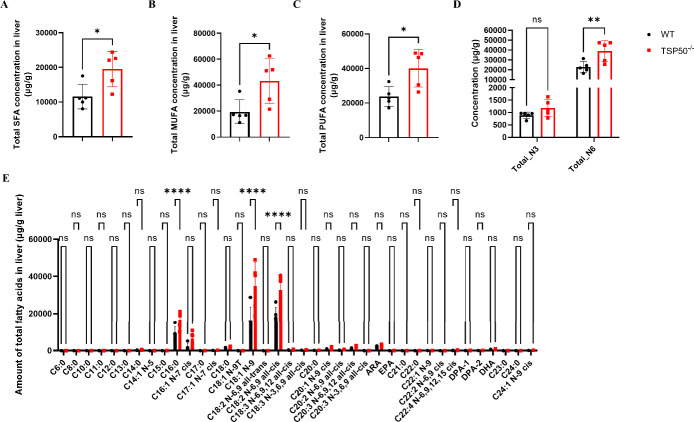
TSP50 deficiency modifies the composition of hepatic fatty acids. **A** Total saturated fatty acids (SFAs) content in the liver of MASH mice in each group. **B** Total monounsaturated fatty acids (MUFAs) content in the liver of MASH mice in each group. **C** Total polyunsaturated fatty acids (PUFAs) content in the liver of MASH mice in each group. **D** Total n-3 PUFAs and n-6 PUFAs content in the liver of MASH mice in each group. **E** Hepatic fatty acid composition in the liver of MASH mice in each group

Based on the observation that TSP50 knockout mice exhibited abnormal hepatic lipid accumulation, particularly elevated TG levels during MASLD progression, we delved deeper into how TSP50 impacts the composition of hepatic fatty acids. It was recently reported that changes in the fatty acid content in the liver contribute to the exacerbation of MASLD and insulin resistance [34, 35]. To address this issue, we extracted the total lipids in the liver and measured the content of the individual fatty acids using gas chromatography–mass spectrometry (GC–MS). We found that TSP50 deficiency led to an increase in SFAs, MUFAs, and polyunsaturated fatty acids (PUFAs) in the liver of MASLD mice (Figs. 4A–C). In the livers of MASH mice, TSP50 deficiency led to a significant increase in omega-6 (N-6) PUFAs, whereas the levels of omega-3 (N-3) PUFAs were not significantly altered (Fig. 4D). An analysis of the fatty acid content in the livers of MASH mice revealed that TSP50 knockout resulted in elevated levels of C16:0 (palmitic acid), C18:1(oleic acid), and C18:2(linoleic acid), which are involved in the induction of oxidative stress, inflammation, and lipid accumulation [36, 37] (Fig. 4E).

### TSP50 suppresses lipid accumulation in hepatocytes

**Fig. 5:**
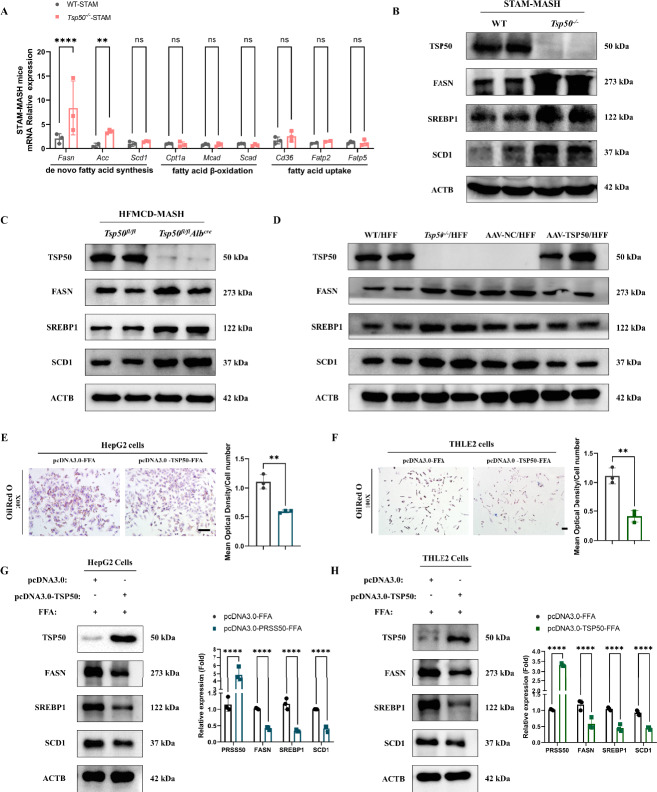
TSP50 suppresses lipid accumulation in hepatocytes. **A** Relative mRNA levels of lipid metabolism genes in livers from STAM/MASH-WT and STAM/MASH-*Tsp50*^*−/−*^ mice. **B** Western blot analysis of de novo lipogenesis (DNL) proteins in liver tissue from STAM/MASH-WT and STAM/MASH-*Tsp50*^*−/−*^ mice-. **C** Western blot results of DNL proteins in liver tissue of the *Tsp50*^*fl/fl*^ and *Tsp50*^*fl/fl*^*Alb*^*Cre*^ mice following the induction of MASH by HFMCD. **D** Western blot results of DNL proteins in liver tissue of the WT, *Tsp50*^*−/−*^, AAV-NC-*Tsp50*^*−/−*^ and AAV-TSP50-*Tsp50*^*−/−*^ mice following the induction of MASH by HFF. **E** HepG2 cells transfected with pcDNA3.0 or pcDNA3.0-TSP50 were treated with free fatty acid (FFA). Lipid accumulation was assessed by Oil Red O staining (left) and quantified (right). Scale bars, 20 μm. **F** THLE2 cells transfected with pcDNA3.0 or pcDNA3.0-TSP50 were treated with FFA. Lipid accumulation was assessed by Oil Red O staining (left) and quantified (right). Scale bars, 20 μm. **G** Western blot analysis of DNL proteins in HepG2 cells after FFA treatment. **H** Western blot analysis of DNL proteins in THLE2 cells after FFA treatment

We next investigated the mechanism by which TSP50 prevents lipid accumulation in the liver. There are mainly three pathways involved in the accumulation of lipid in the liver: 1) fatty acid uptake, 2) fatty acid synthesis, 3) fatty acids β-oxidation. We first examined the mRNA levels of lipid metabolism-related genes in the liver tissues of STAM/MASH mice and found that the mRNA levels of genes related to DNL were obviously upregulated in *Tsp50*^*−/−*^ mice compared with WT mice (Fig. 5A). However, no significant differences were observed in genes related to fatty acid β-oxidation and fatty acid uptake between the two groups. Western blot analyses further confirmed that the expression of proteins involved in DNL including FASN, SREBP1c, and SCD1 was elevated in the liver tissues of *Tsp50*^*−/−*^ mice during MASLD progression (Figs. 5B, S4A, S4B). Similar findings were observed in *Tsp50*^*fl/fl*^*Alb*^*Cre*^ mice subjected to a HFMCD diet and in the liver tissues of *Tsp50*^*−/−*^mice fed a HFF diet (Fig. 5C). Notably, AAV-mediated restoration of TSP50 expression in *Tsp50*^*−/−*^ mice significantly reduced the expression of these lipid synthesis proteins (Fig. 5D). Furthermore, we also examined hepatic TSP50 expression in wild-type normal mice and wild-type MASH mice. Immunohistochemistry and western blot results revealed that TSP50 protein levels were significantly elevated in the MASH model, indicating its upregulation in MASLD (Supplementary Figs. S4C, S4D).

To investigate the mechanism by which TSP50 influences lipid metabolism in hepatocytes, we overexpressed Flag-tagged TSP50 (Flag-TSP50) in hepatocytes. The capacity of TSP50 to metabolize lipids was subsequently assessed by introducing free fatty acids (FFA) into the medium. The Oil Red O staining demonstrated that the hepatocyte lipid accumulation induced by FFA was significantly alleviated in the TSP50 overexpression group compared with the control group (Figs. 5E, F). Consistent with these findings, intracellular TG levels were decreased in TSP50-overexpressing hepatocytes, while TCHO levels remained unchanged (Supplementary Figs. S4E, S4F). In addition, the mRNA expression levels of lipid metabolism-related proteins were altered in TSP50-overexpressing hepatocytes (Supplementary Fig. S4G). The western blot analysis revealed a decreased expression of proteins involved in de novo lipid synthesis following the overexpression of TSP50 (Figs. 5G, 5H). Collectively, these results indicated that TSP50 plays a critical role in suppressing lipid accumulation in hepatocytes by modulating de novo lipid synthesis pathways.

### TSP50 directly binds to SCD1 and degrades it through a catalytic triad structure

**Fig. 6:**
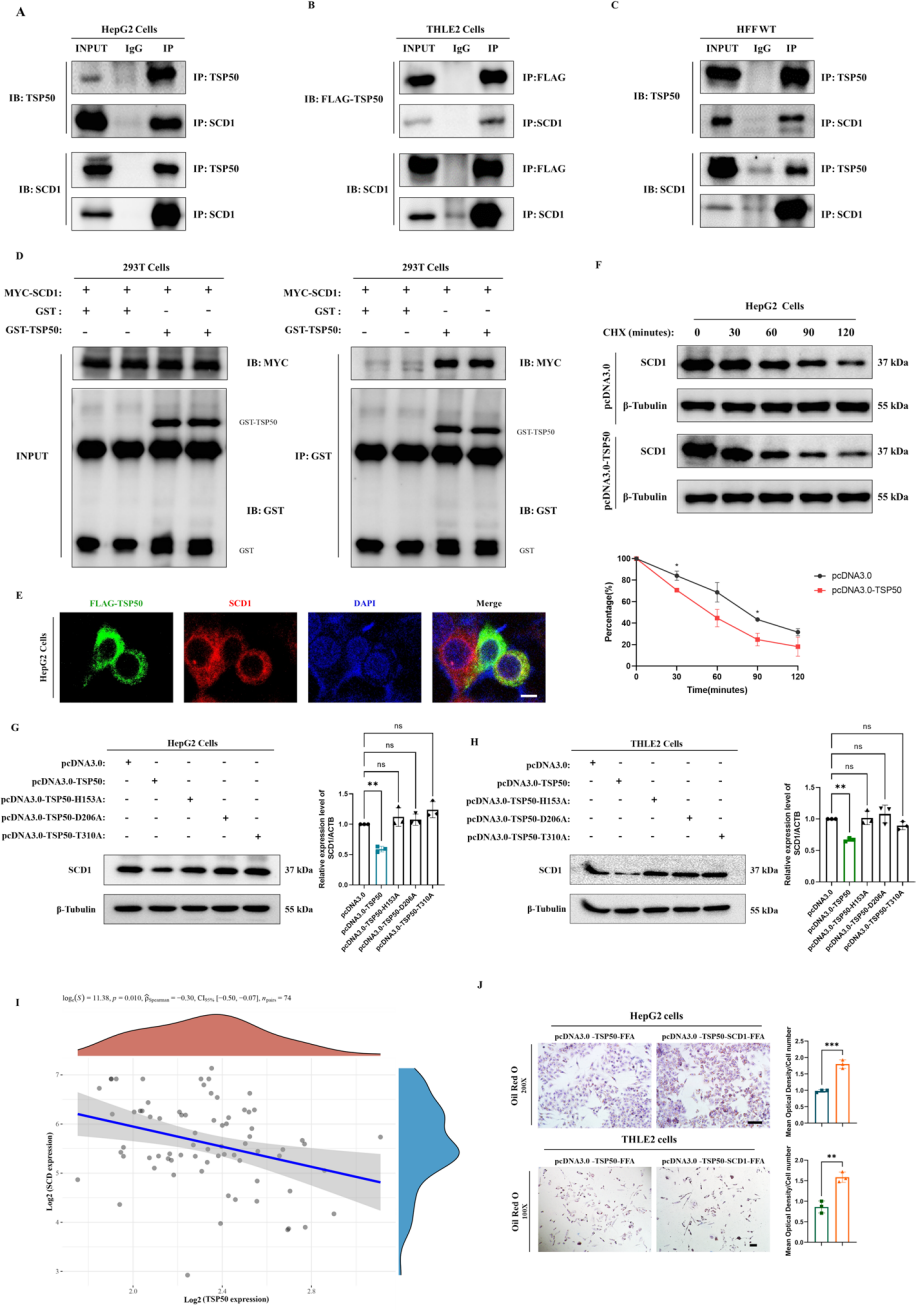
TSP50 directly binds to SCD1 and degrades it through a catalytic triad structure. **A** Co-IP and western blot analysis of the interaction between TSP50 and SCD1 in the HepG2 cells. **B** Co-IP and western blot analysis of the interaction between TSP50-FLAG and SCD1 in the THLE2 cells. **C** Co-IP and western blot analysis of the interaction between TSP50 and SCD1 in the livers of HFF/MASH-WT mice. **D** GST pull-down and western blot analysis of the interaction between TSP50-GST and SCD1-MYC proteins. **E** Immunofluorescence staining was performed to visualize the colocalization of TSP50-FLAG and SCD1 in HepG2 cells. **F** The protein levels of SCD1 in HepG2 cells transfected with pcDNA3.0-TSP50 or the corresponding empty vector control were treated with 1 mM CHX. **G** The levels of SCD1 protein in HepG2 cells overexpressing wild-type TSP50 and three different TSP50 mutants. **H** The levels of SCD1 protein in THLE2 cells overexpressing wild-type TSP50 and three different TSP50 mutants. **I** Correlation analysis of TSP50 and SCD1 in liver tissue from patients with MASH. **J** HepG2 and THLE2 cells were transfected with pcDNA3.0-TSP50 or cotransfected with pcDNA3.0-TSP50 and pcDNA3.0-SCD1, followed by treatment with FFA. Lipid accumulation was visualized using Oil Red O staining (Left) and the results were quantified (Right). Scale bars, 20 μm

To elucidate how TSP50 regulates the expression of DNL genes, we investigated its interaction with key proteins involved in lipid synthesis. Co-IP experiments demonstrated that TSP50 interacts exclusively with SCD1, rather than other lipid synthesis-related proteins, in HepG2 cells (Supplementary Figs. S5A, 6A, 6B). This specific interaction was further confirmed by cotransfecting HepG2 cells with FLAG-tagged TSP50 and MYC-tagged SCD1, followed by Co-IP assays, which showed the formation of a complex between FLAG-TSP50 and MYC-SCD1 (Supplementary Fig. S5B). In addition, validation experiments conducted with the liver tissue of HFF-induced MASH mice also demonstrated the interaction between TSP50 and SCD1 (Figs. 6C, D).

To further investigate the direct interaction between TSP50 and SCD1, we performed GST pull-down assays using purified GST-TSP50 expressed in *E. coli* BL21. The results showed that TSP50 could directly bind to SCD1 (Fig. 6E). Meanwhile, immunofluorescence was utilized to assess the colocalization of TSP50 and SCD1 within hepatocytes. Consistent with the Co-IP results, the expression patterns of TSP50 and SCD1 overlapped in hepatocytes. Altogether, these data suggest that TSP50 can interact with SCD1.

Since TSP50 is a serine protease and lipid synthesis is significantly increased after TSP50 knockout, we next investigated the potential role of TSP50 in modulating the stability of SCD1 protein. Overexpression of TSP50 in hepatocytes led to a significant acceleration in the rate of SCD1 protein degradation in the presence of cycloheximide (CHX) treatment, suggesting that TSP50 promotes the degradation of SCD1 (Fig. 6F). However, TSP50 did not affect the ubiquitination of SCD1, as confirmed by treatment with the proteasome inhibitor MG132 (Supplementary Fig. S5C), indicating that TSP50-mediated SCD1 degradation likely occurs through a ubiquitin-independent pathway.

To investigate the dependence of SCD1 degradation on the hydrolase activity of TSP50, we overexpressed a mutant form of TSP50 lacking its catalytic triad structure in hepatocytes. The findings indicated that the catalytic triad structure of TSP50 was essential for its ability to degrade SCD1 (Fig. 6G, H). To assess the clinical significance of our findings, we examined the correlation between TSP50 and SCD1 expression levels in liver tissues from patients with MASH from the publicly available transcriptomic dataset Gene Expression Omnibus (GEO: Profile # GSE164760). The results showed that TSP50 was negatively correlated with SCD1 in liver tissues of patients with MASH, supporting the notion that TSP50 downregulates SCD1 in the context of MASH (Fig. 6I).

To assess the functional significance of TSP50-mediated SCD1 degradation, we overexpressed both TSP50 and SCD1 in cells and treated them with FFA to induce lipid accumulation. The findings suggest that the inhibitory effect of TSP50 overexpression on lipid accumulation was reversed by the overexpression of SCD1 (Fig. 6J), highlighting the critical role of TSP50 in regulating lipid metabolism via SCD1 degradation.

Overall, these findings indicate that TSP50 directly interacts with SCD1 and degrades it through its catalytic triad structure, thereby suppressing lipid accumulation in hepatocytes.

### SCD1 inhibition reverses TSP50 deficiency-induced MASLD in mice

**Fig. 7:**
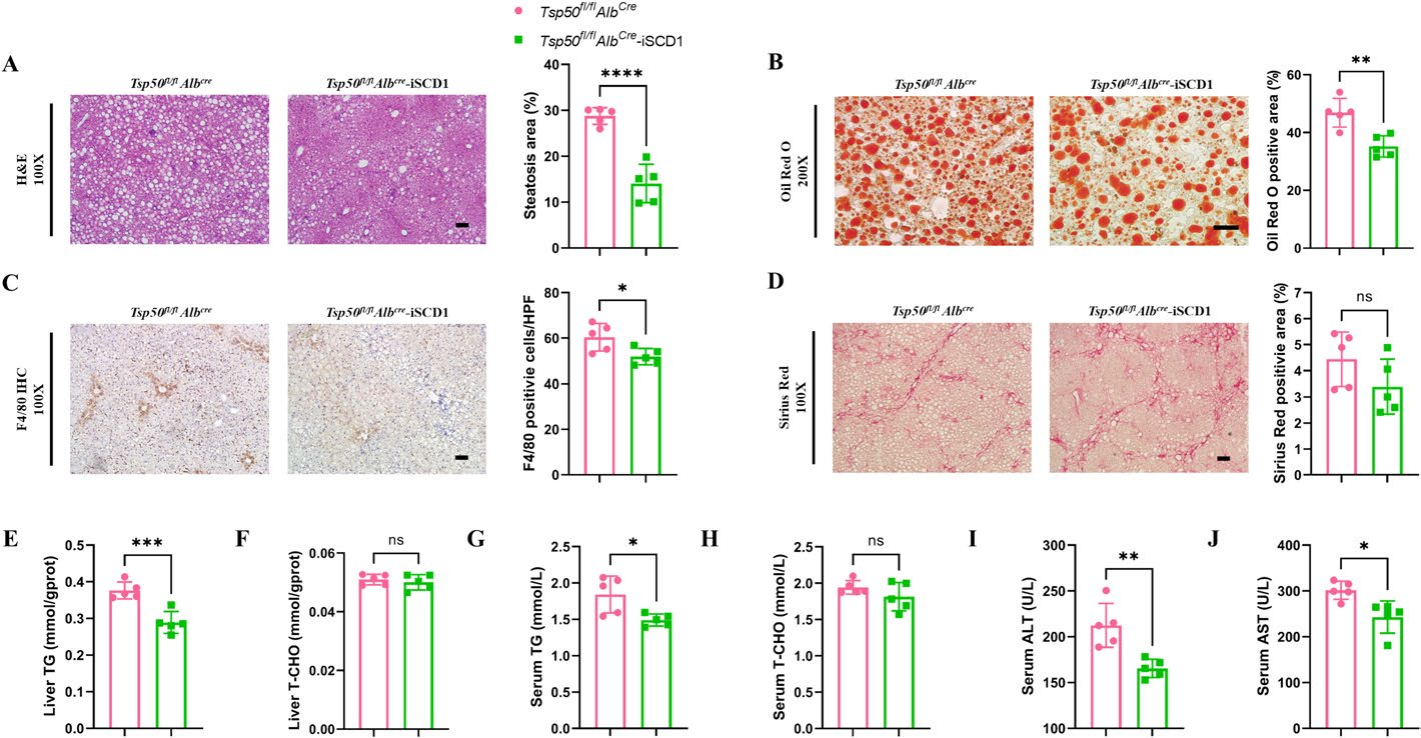
SCD1 inhibition reverses TSP50 deficiency-induced MASLD in mice. **A** H&E staining (left) and steatosis quantification (right) in liver sections from *Tsp50*^*fl/fl*^*Alb*^*Cre*^ and *Tsp50*^*fl/fl*^*Alb*^*Cre*^-iSCD1 mice (*n* = 5 mice/group). Scale bars, 20 μm. **B** Oil Red O staining (left) and quantification (right) in liver sections from *Tsp50*^*fl/fl*^*Alb*^*Cre*^ and *Tsp50*^*fl/fl*^*Alb*^*Cre*^-iSCD1 mice (*n* = 5 mice/group). Scale bars, 20 μm. **C** F4/80 IHC positive area quantification in liver sections from *Tsp50*^*fl/fl*^*Alb*^*Cre*^ and *Tsp50*^*fl/fl*^*Alb*^*Cre*^-iSCD1 mice (*n* = 5 mice/group). Scale bars, 20 μm. **D** Sirius Red staining (left) and quantification (right) in liver sections from *Tsp50*^*fl/fl*^*Alb*^*Cre*^ and *Tsp50*^*fl/fl*^*Alb*^*Cre*^-iSCD1 mice (*n* = 5 mice/group). The Sirius Red-positive area in each area range was quantified by ImageJ Pro Plus software. Scale bars, 20 μm. **E** Hepatic TG content of *Tsp50*^*fl/fl*^*Alb*^*Cre*^ and *Tsp50*^*fl/fl*^*Alb*^*Cre*^-iSCD1 mice (*n* = 5 mice/group). **F** Hepatic TCHO content of*Tsp50*^*fl/fl*^*Alb*^*Cre*^ and *Tsp50*^*fl/fl*^*Alb*^*Cre*^-iSCD1 mice (*n* = 5 mice/group). **G** Serum TG content of *Tsp50*^*fl/fl*^*Alb*^*Cre*^ and *Tsp50*^*fl/fl*^*Alb*^*Cre*^-iSCD1 mice (*n* = 5 mice/group). **H** Serum TCHO content of *Tsp50*^*fl/fl*^*Alb*^*Cre*^ and *Tsp50*^*fl/fl*^*Alb*^*Cre*^-iSCD1 mice (*n* = 5 mice/group). **I** Serum ALT level of *Tsp50*^*fl/fl*^*Alb*^*Cre*^ and *Tsp50*^*fl/fl*^*Alb*^*Cre*^-iSCD1 mice (*n* = 5 mice/group). **J** Serum AST level of *Tsp50*^*fl/fl*^*Alb*^*Cre*^ and *Tsp50*^*fl/fl*^*Alb*^*Cre*^-iSCD1 mice (*n* = 5 mice/group)

To investigate the potential involvement of TSP50 in the progression of MASLD through the regulation of SCD1 in vivo, we selected MK-8245, a liver-targeted inhibitor of SCD1, for oral treatment of *Tsp50*^*fl/fl*^*Alb*^*Cre*^ mice fed an HFMCD diet. Our results indicated that, compared with other experimental groups, mice treated with MK-8245 exhibited more pronounced weight loss during MASH progression, although no significant differences in liver-to-body weight ratios were observed among the groups (Supplementary Fig. S6A). Histological analyses revealed that MK-8245 treatment significantly alleviated hepatic lipid accumulation, steatosis, inflammation, and fibrosis in these mice (Figs. 7A–D). Biochemical assays further demonstrated that MK-8245 treatment led to substantial reductions in hepatic and serum TG levels, while hepatic and serum TCHO levels remained unchanged (Figs.7E–H). In addition, the serum levels of ALT and AST were significantly decreased in mice treated with MK-8245 (Figs. 7I, J). Altogether, these results suggest that deletion of TSP50 in hepatocytes promotes MASLD formation in mice and this effect is at least partially dependent on SCD1. Overall, these data suggest that the inhibitor of SCD1 rescued hepatic TSP50 knockout induced lipid accumulation and liver injury during MASLD.

## Discussion

MASLD is a complex, multifactorial disorder characterized by dysregulated hepatic lipid metabolism. Accumulating evidence indicates that increased hepatic DNL is a pivotal driver of MASLD progression in both rodents and humans [38–42]. Using diet-induced mouse models, our present study demonstrates that the loss of TSP50 drives MASLD pathogenesis by promoting lipid accumulation, uncovering a novel axis connecting TSP50 to SCD1 and DNL.

During MASLD progression, the key enzymes involved in DNL are significantly upregulated, which contributes to excessive total hepatic lipid accumulation and metabolic dysfunction [43, 44]. Consequently, inhibitors targeting ACC, FASN, and SCD1 have been developed as drug candidates and are currently in clinical testing for MASLD therapy [45]. Meanwhile, research has revealed some novel regulators of DNL pathway that are involved in hepatic lipid reprogramming and MASLD pathogenesis, presenting new opportunities for targeted therapies. For instance, tripartite motif-containing 21 (TRIM21) was induced in livers of humans and mice with MASLD and TRIM21 overexpression inhibited the accumulation of hepatic triglycerides by ubiquitinating and degrading multiple key proteins during metabolic stress [46]. In addition, the adhesion G protein-coupled receptor F1(ADGRF1) is downregulated during MASLD progression and Adgrf1 overexpression aggravated the severity of obesity-induced MASLD by enhancing the transcription of SCD1 and liver steatosis. Thus, the downregulation of hepatic Adgrf1 expression level likely represented a protective mechanism against over-accumulation of lipid and disease progression [47].

As reported, diet-induced hepatic DNL in mice is predominantly regulated by SCD1 [48]. SCD1 is the key enzyme in triglyceride biosynthesis, introducing a delta-9 double bond into saturated fatty acids (C16:0 and C18:0) to produce MUFAs (C16:1 and C18:1) [49]. When fructose was supplemented in the diet of *SCD1*^*−/−*^ mice, it failed to upregulate DNL related enzymes such as SREBP1, FASN, and ACC. Dietary supplementation with MUFAs increased SREBP1 transcription and enhanced hepatic DNL. In contrast, dietary supplementation with SFAs or PUFAs could not promote hepatic DNL of *SCD1*^*−/−*^ mice. Furthermore, in *SREBP1*^*−/−*^ mice, dietary addition of fructose still upregulated other key enzymes involved in DNL, and the addition of MUFAs to the diet further stimulated the process of DNL [16].These findings underscore the pivotal role of SCD1 in hepatic DNL. Both pharmacological inhibition and antisense oligonucleotide (ASO)-mediated suppression of SCD1 effectively attenuate hepatic DNL and show therapeutic potential in MASLD models [15, 50–52]. In this present study, we demonstrate for the first time that the protease activity of TSP50, an upstream regulator of SCD1, is crucial for modulating the stability of SCD1 and consequently regulating hepatic lipid synthesis.

Given the biological function of SCD1, the fatty acid content including MUFAs, the enzymatic product of SCD1, needs to be further investigated. TSP50 knockout increased the levels of C16:0, C18:1, and C18:2. The amounts of octadecenoic acid (C18:1) were most dynamically upregulated, implying that TSP50 may be involved in regulating SCD1 activity. Previous studies have shown that MUFA reduces lipotoxicity compared to SFAs [53], suggesting a potentially beneficial effect on MASLD. However, MUFA generated by SCD1 facilitate the accumulation of TGs within cells [54, 55], and this excessive accumulation of TGs contributes to the progression of MASLD [56]. In our study, we found that the aberrant elevation of SCD1 in the livers of MASH mice following TSP50 knockout significantly accelerated the rate of lipid accumulation during the progression of MASLD, thereby exacerbating the disease progression. The contradictory roles of SCD1 and its derived MUFAs in MASLD highlight their context-dependent actions. Early-stage MASLD may benefit from MUFA-mediated SFA replacement, reducing lipotoxicity, while chronic progression leads to lipid overload and toxicity, as shown in our work. As recently reported, liver-specific SCD1 deficiency induces endoplasmic reticulum (ER) stress via mTORC1 activation in global knockout models, and oleate paradoxically suppresses mTORC1 signaling and alleviates ER stress [57]. Similarly, SCD1-mediated lipid desaturation has been shown to promote ER stress in hepatocellular carcinoma [58]. Therefore, therapeutic strategies targeting SCD1 require further experimental validation to assess their efficacy. Here, our findings in TSP50-deficient mice suggest that under conditions of chronic metabolic stress, particularly in advanced MASLD models mimicking human disease progression, the sustained overproduction of MUFAs by hyperactive SCD1 drives excessive triglyceride synthesis and lipid droplet expansion that overwhelms the liver’s lipid disposal capacity. Accordingly, subsequent intervention with an SCD1 inhibitor reversed the lipid accumulation in the livers of MASLD mice.

Previous studies have focused on the involvement of TSP50 in tumorigenesis in multiple cancer types. Abnormal expression or activation of TSP50 is associated with poor prognosis in breast cancer [59, 60], colorectal cancer [61], nonsmall cell lung cancer [62], gastric cancer [29], and hepatocellular carcinoma (HCC) [25, 26]. In breast cancer, TSP50 promotes cell proliferation by suppressing activin signaling through its interaction with ActRIIA, which inhibits the ActRIB/Smad2/3 pathway [63]. Moreover, TSP50 promotes cell proliferation, invasion, and metastasis at least partially via nuclear factor (NF)-κB pathway activation in breast cancer cells [64] and gastric cancer cells [65]. TSP50 also enhances cancer stem cell (CSC)-like properties and epithelial-mesenchymal transition (EMT) in breast cancer cells by interacting with PI3K p110α to promote p110α enzymatic activity and activate the PI3K/AKT pathway [60]. Similarly, this pro-tumorigenic mechanism was found in colorectal cancer cells [66]. Furthermore, TSP50 drives metabolic reprogramming in HCC by binding to and promoting the acetylation of key metabolic enzymes PKM2 [25] and G6PD [26], ultimately promoting tumor cell proliferation and tumorigenesis. Different from prior reports, our study reveals that global deletion of TSP50 accelerates tumor development in the STAM mouse model, suggesting a protective function for TSP50 in MASLD-related HCC. However, apart from the established role of TSP50 in DNL, the precise function and mechanism of TSP50 in HCC, particularly in MASLD-HCC, remain unclear. Given that findings from global knockout models are potentially confounded by developmental compensation and non-cell-autonomous effects, further studies utilizing a hepatocyte-specific TSP50 knockout model are essential to elucidate its role in MASLD-HCC. Furthermore, metabolic stress, such as a high-lipid environment, is likely to modulate the function of TSP50 in cancer development. Thus, as a protease, several key questions regarding TSP50 remain to be addressed: whether its expression and activity undergo dynamic changes during MASLD and cancer progression, whether these changes are linked to systemic metabolic stress, and whether its oncogenic role is directly dependent on its protease activity in different cancers. Investigating these points will provide valuable insights for developing TSP50-targeted therapies.

Several limitations of our study should be acknowledged. First, the main experiments were conducted in vitro and mouse models. The expression of TSP50 and its interaction with SCD1 in human MASLD/HCC tissues require further investigation to determine the translational potential. Second, although our therapeutic strategies by TSP50 overexpression and SCD1 inhibitors showed efficacy in MASLD models, the long-term safety of AAV-mediated TSP50 overexpression or SCD1 inhibition has not been assessed. Accordingly, targeted therapy against TSP50 requires careful evaluation in the future. Finally, our work is currently restricted to male mice and it is unknown whether the described mechanisms operate similarly in females, given the known sexual dimorphism in liver diseases.

In conclusion, our findings revealed that TSP50 is involved in the hepatic lipid accumulation by regulating SCD1 during the development of MASLD, providing novel mechanistic insight into MASLD pathogenesis and new ideas for developing therapeutic strategies.

## Supplementary Information

Supplementary Material 1

## Data Availability

The data that support the findings of this study are available from the corresponding author upon reasonable request.

## Abbreviations

TSP50: Testes-specific protease 50
MASLD: Metabolic dysfunction-associated steatotic liver disease
NAFLD: Non-alcoholic fatty liver disease
STAM: STelic Animal Model
HCC: Hepatocellular carcinoma
MASH: Metabolic dysfunction-associated steatohepatitis
NASH: Non-alcoholic steatohepatitis
SCD1: Stearoyl-CoA desaturase 1
DNL: De novo lipogenesis
SFAs: Saturated fatty acids
MUFAs: Monounsaturated fatty acids
PUFAs: Polyunsaturated fatty acids
siRNA: Small interfering RNA
G6PD: Glucose-6-phosphate dehydrogenase
PKM2: Pyruvate kinase M2
SPF: Specific pathogen-free
WT: Wild-type
HFF: High-fat/high-cholesterol plus high fructose
HFMCD: High-fat with methionine and choline deficiency
MASL: Metabolic dysfunction-associated steatotic liver
AFP: Alpha-fetoprotein
AAV: Adeno-associated virus
CO-IP: Co-immunoprecipitation
ADGRF1: Adhesion G protein-coupled receptor F1
TRIM21: Tripartite motif containing 21
SNX8: Sorting nexin 8
FASN: Fatty acid synthase
ASO: Antisense oligonucleotide
