# Supplementary material for: TSP50 attenuates metabolic dysfunction-associated steatotic liver disease via SCD1 degradation-mediated suppression of hepatocyte lipogenesis
Source: Cell Mol Biol Lett. 2026 Feb 1;31:20. doi: 10.1186/s11658-026-00859-2 (PMC12874672; doi:10.1186/s11658-026-00859-2)
Supplement: Supplementary file 1 — Supplementary Material 1 [file 11658_2026_859_MOESM1_ESM.docx]

# S**upplementary Text**

Supplementary material contains supplementary images (Fig. S1-S6) mentioned in the main text, and other experimentally relevant supplementary information.

## Fig. S1. Systemic TSP50 deficiency exacerbates MASLD progression to HCC.


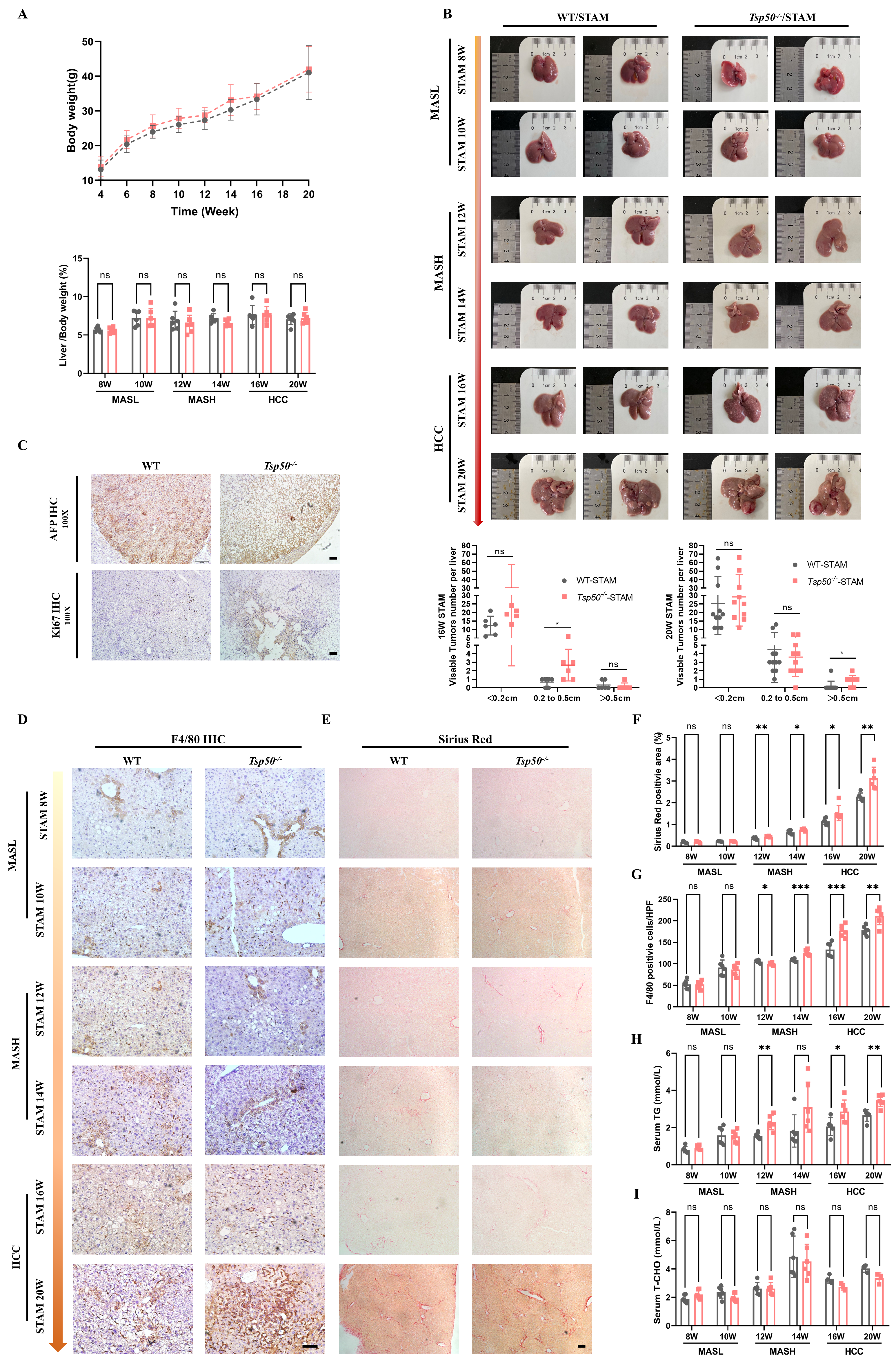


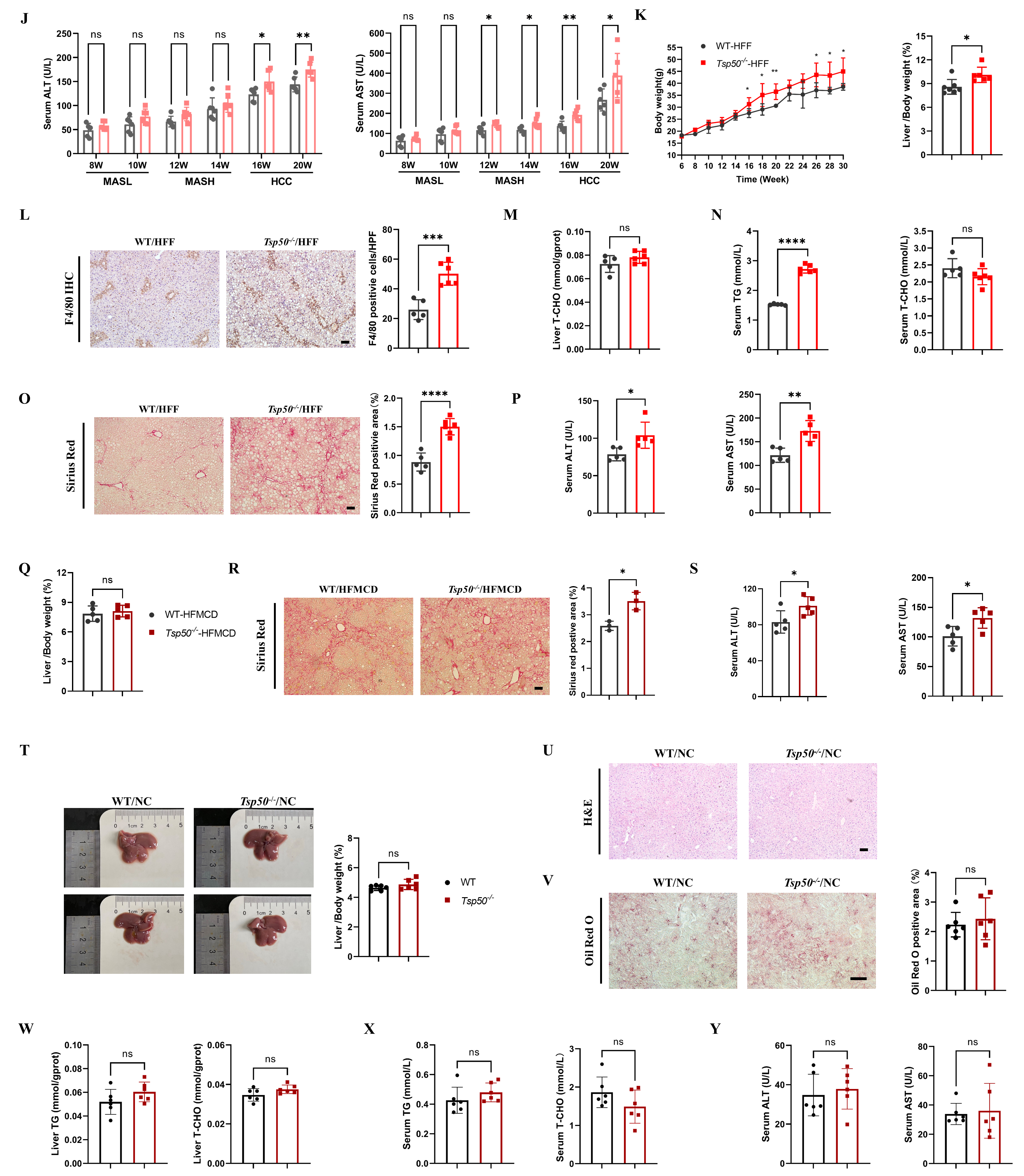


1. Body weight trajectories and liver/body weight ratio of STAM-WT and STAM-*Tsp50^-/-^* mice.
2. Liver appearance and number of visible nodules of STAM-WT and STAM-*Tsp50^-/-^* mice.
3. AFP IHC and Ki67 IHC staining in liver sections from STAM-WT and STAM-*Tsp50^-/-^* mice Scale bars, 20 μm.
4. F4/80 IHC staining in liver sections from STAM-WT and STAM/MASLD-*Tsp50^-/-^* mice (n = 6 mice/group). Scale bars, 20 μm.
5. Sirius Red staining in liver sections from STAM-WT and STAM/MASLD-*Tsp50^-/-^* mice (n = 6 mice/group). Scale bars, 20 μm.
6. F4/80 IHC positive area quantification in liver sections from STAM-WT and STAM-*Tsp50^-/-^* mice (n = 6 mice/group).
7. Sirius Red positive area quantification in liver sections of STAM-WT and STAM-*Tsp50^-/-^* mice (n = 6 mice/group).
8. Serum TG content of STAM-WT and STAM-*Tsp50^-/-^* mice (n = 6 mice/group).
9. Serum TCHO content of STAM-WT and STAM-*Tsp50^-/-^* mice (n = 6 mice/group).
10. Serum ALT and AST level of STAM-WT and STAM-*Tsp50^-/-^* mice (n = 6 mice/group).
11. Body weight trajectories and liver/body weight ratio of HFF/MASH-WT and HFF/MASH-*Tsp50^-/-^* mice.
12. F4/80 IHC staining (left) and steatosis quantification (right) in liver sections from HFF/MASH-WT and HFF/MASH-*Tsp50^-/-^* mice (n = 5 mice/group). Scale bars, 20 μm.
13. Hepatic TCHO content of HFF/MASH-WT and HFF/MASH-*Tsp50^-/-^* mice (n = 5 mice/group).
14. Serum TG and TCHO content of HFF/MASH-WT and HFF/MASH-*Tsp50^-/-^* mice (n = 5 mice/group).
15. Sirius Red staining (left) and quantification (right) in liver sections from HFF/MASH-WT and HFF/MASH-*Tsp50^-/-^* mice (n = 5 mice/group). Scale bars, 20 μm.
16. Serum ALT and AST level of HFF/MASH-WT and HFF/MASH-*Tsp50^-/-^* mice (n = 5 mice/group).
17. Liver/body weight ratio of HFMCD/MASH-WT and HFMCD/MASH-*Tsp50^-/-^* mice.
18. Sirius Red staining (left) and quantification (right) in liver sections from HFMCD/MASH-WT and HFMCD/MASH-*Tsp50^-/-^* mice (n = 5 mice/group). Scale bars, 20 μm.
19. Serum ALT and AST level of HFF/MASH-WT and HFF/MASH-*Tsp50^-/-^* mice (n = 5 mice/group).
20. Liver Appearance and liver/body weight ratio of WT and *Tsp50^-/-^* mice.
21. H&E staining in liver sections from WT and *Tsp50^-/-^* mice (n = 5 mice/group). Scale bars, 20 μm.
22. Oil Red O staining (left) and quantification (right) in liver sections from WT and *Tsp50^-/-^* mice (n = 5 mice/group). Scale bars, 20 μm.
23. Hepatic TG and TCHO content of WT and *Tsp50^-/-^* mice (n = 5 mice/group).
24. Serum TG and TCHO content of WT and *Tsp50^-/-^* mice (n = 5 mice/group).
25. Serum ALT and AST level of WT and *Tsp50^-/-^* mice (n = 5 mice/group).

## Fig S2. TSP50 deficiency in hepatocyte accelerates MASLD/MASH progression.

**
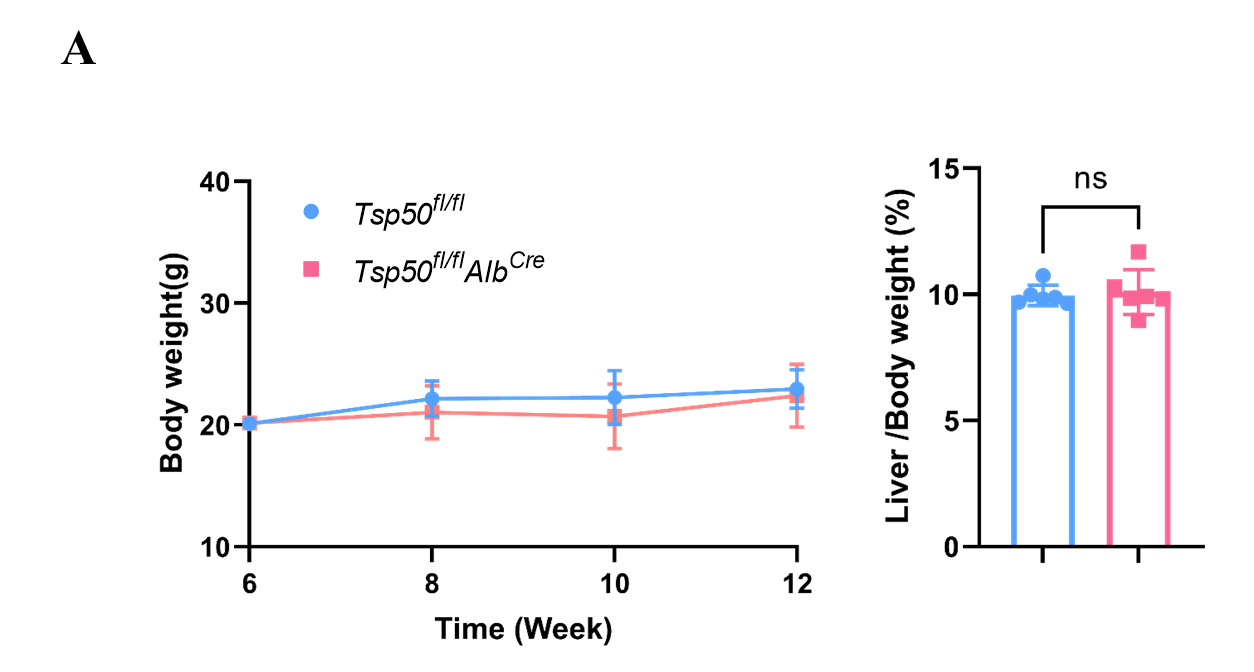
**

1. Body weight trajectories and liver/body weight ratio of HFF/MASH-WT and HFF/MASH-*Tsp50^-/-^* mice.

## Fig S3. Complementation of TSP50 protects against MASLD/MASH progression.


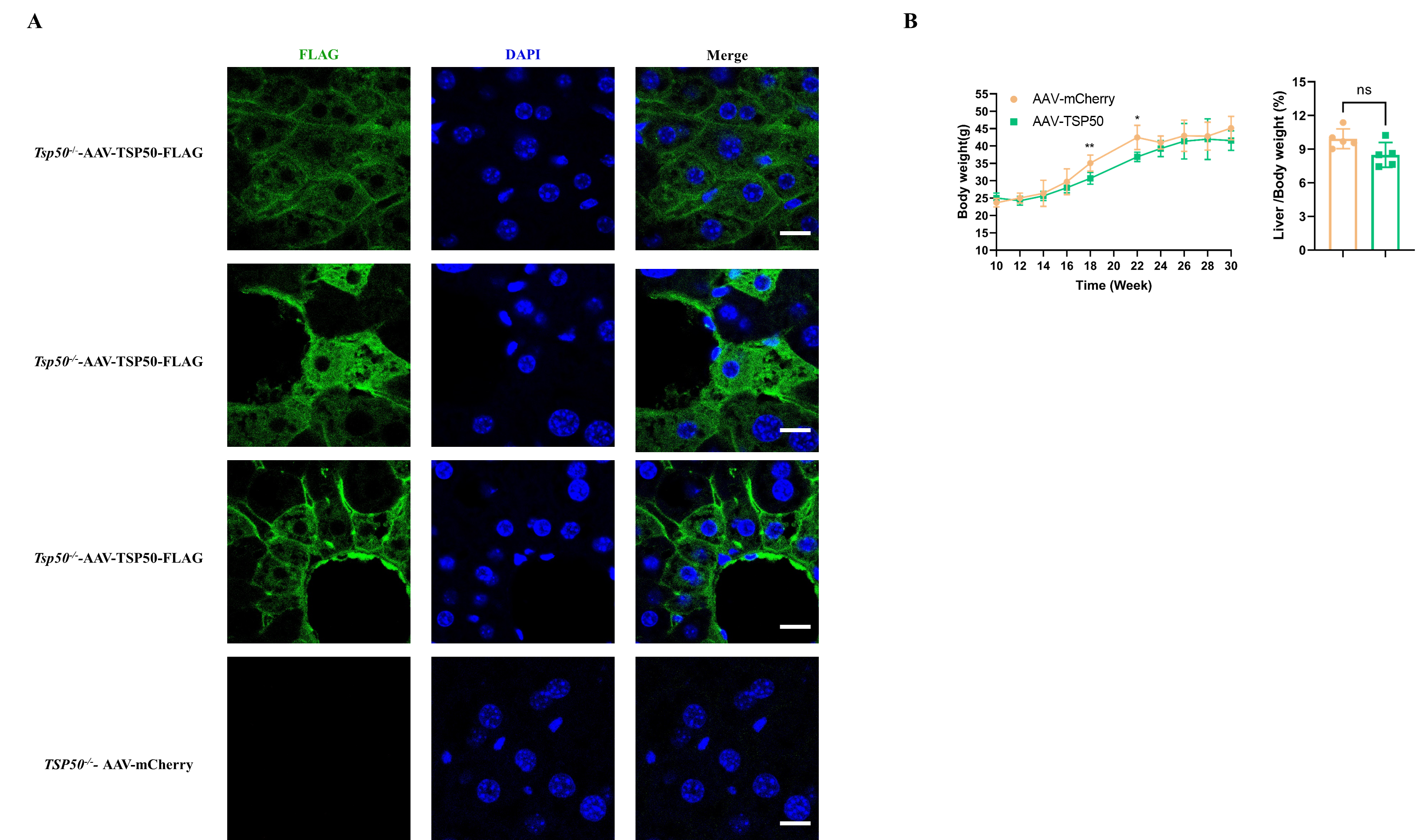


1. Immunofluorescence staining of the liver slides of AAV-mCherry and AAV-TSP50 mice. Scale bars, 20 μm
2. Body weight trajectories and liver/body weight ratio of AAV-mCherry and AAV-TSP50 mice.

## Fig S4. TSP50 suppresses lipid accumulation in hepatocytes.


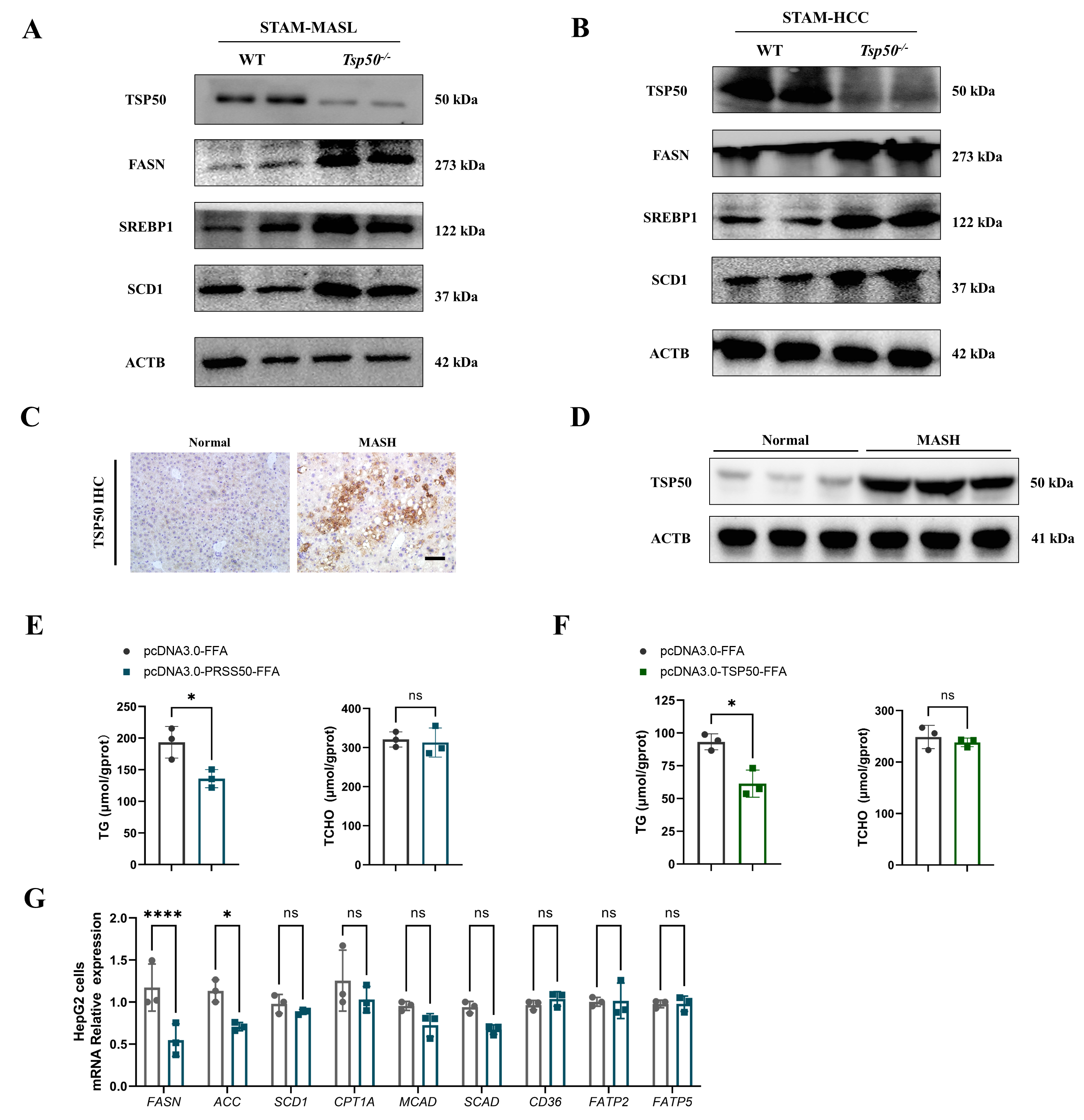


1. WB results of DNL protein in liver tissue of STAM/MASL-WT and STAM/MASL-*Tsp50^-/-^* mice.
2. WB results of DNL protein in liver tissue of STAM/HCC-WT and STAM/HCC-*Tsp50^-/-^* mice.
3. TSP50 IHC staining in liver sections from Normal/MASH-WT mice. Scale bars, 20 μm.
4. WB results of TSP50 in liver tissue of Normal/MASH-WT mice.
5. TG and TCHO content of HepG2 cells in the indicated groups.
6. TG and TCHO content of THLE2 cells in the indicated groups.
7. Relative mRNA levels of lipid metabolism genes in HepG2 cells after FFA consumption.

## Fig S5. TSP50 directly binds to SCD1 and degrades it through a catalytic triad structure.

**
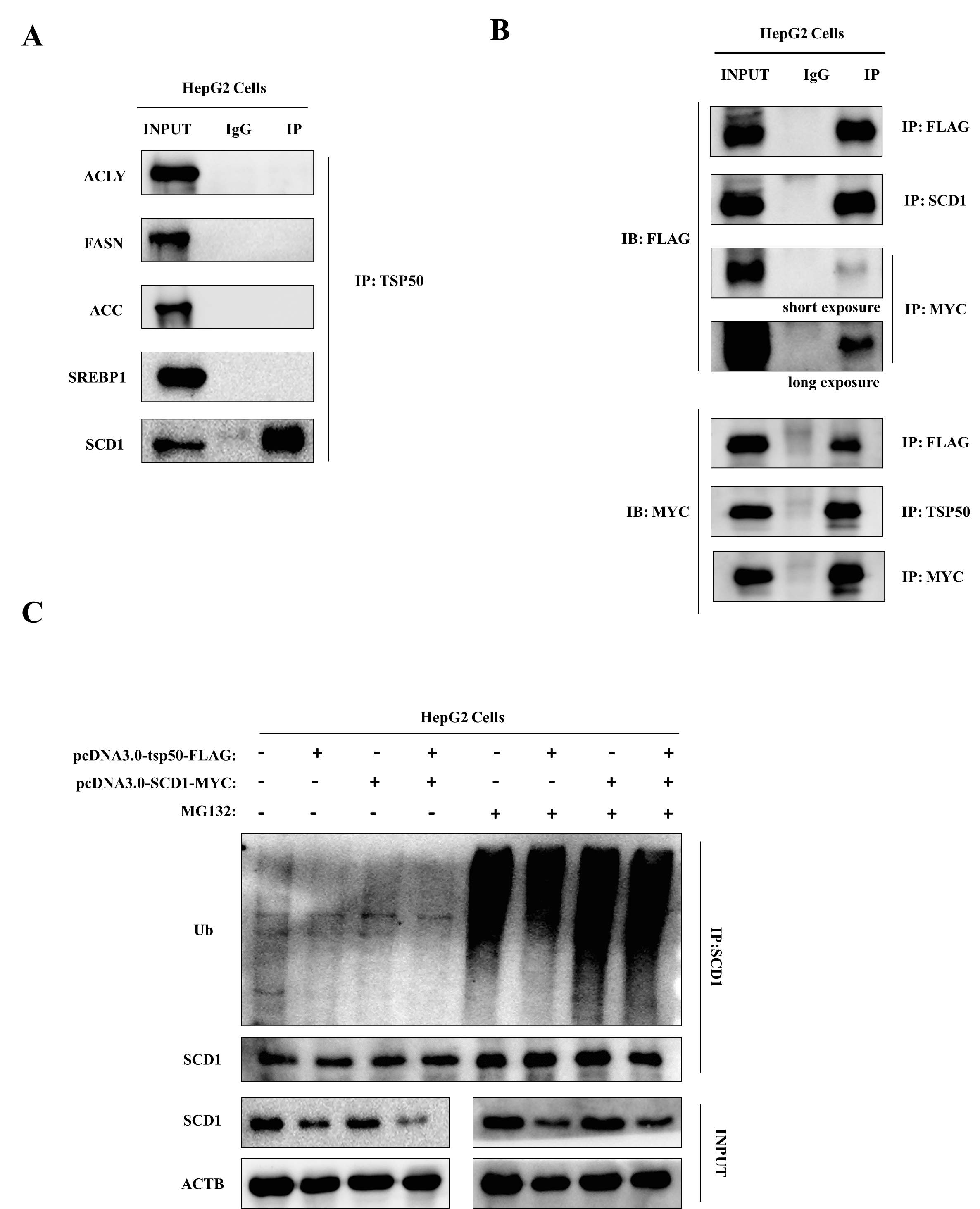
**

1. IP and Western blot analysis of the interaction TSP50 between indicated proteins in the HepG2 cells.
2. IP and Western blot analysis of the interaction TSP50-FLAG between SCD1-MYC proteins in the HepG2 cells.
3. Ubiquitination analysis n HepG2 cells after transfection with pcDNA3.0-TSP50, pcDNA3.0-SCD1 and its corresponding control followed by MG132 treatment.

## Fig S6. SCD1 inhibition reverses TSP50 deficiency-induced MASLD in mice.

**
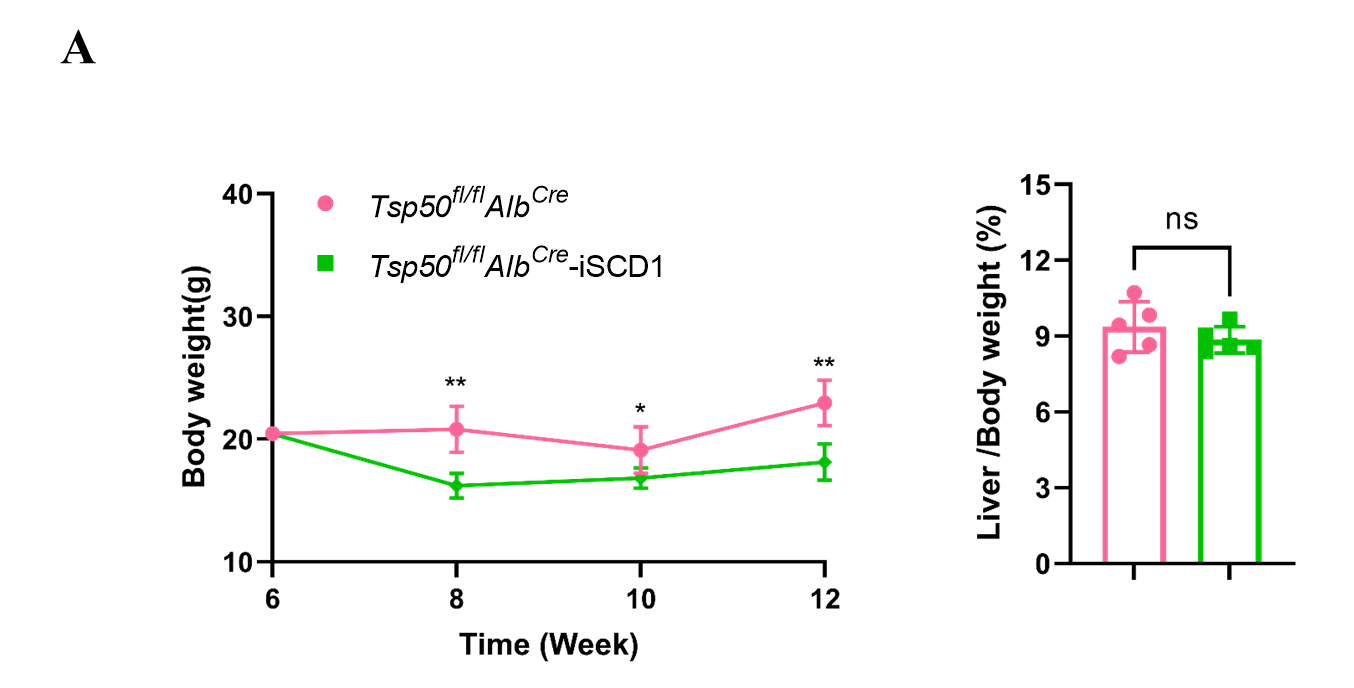
**

1. Body weight trajectories and liver/body weight ratio of HFF/MASH-WT and HFF/MASH-*Tsp50^-/-^* mice.

## Table S1. Sequences of qPCR primers

| Symbol | Primer | Primer Sequence (5′–3′) |
| --- | --- | --- |
| *TSP50*（Human） | *F-Primer* | *TGGAGACCCAATTCCCAGTTT* |
|  | *R-Primer* | *TAACATCACGCCAGATCAGGC* |
| *Tsp50*（Mouse） | *F-Primer* | *ACAGGGAGGAGTTCTGCTATGAGATAAC* |
|  | *R-Primer* | *AAAGATGGGTGGGGCCTCGCTCTTCTTG* |
| *FASN*（Human） | *F-Primer* | *AAGGACCTGTCTAGGTTTGATGC* |
|  | *R-Primer* | TGGCTTCATAGGTGACTTCCA |
| *Fasn*（Mouse） | *F-Primer* | GGAGGTGGTGATAGCCGGTAT |
|  | *R-Primer* | TGGGTAATCCATAGAGCCCAG |
| *ACC*（Human） | *F-Primer* | ATGTCTGGCTTGCACCTAGTA |
|  | *R-Primer* | CCCCAAAGCGAGTAACAAATTCT |
| *Acc*（Mouse） | *F-Primer* | CTCCCGATTCATAATTGGGTCTG |
|  | *R-Primer* | TCGACCTTGTTTTACTAGGTGC |
| *SCD1*（Human） | *F-Primer* | TCTAGCTCCTATACCACCACCA |
|  | *R-Primer* | TCGTCTCCAACTTATCTCCTCC |
| *Scd1*（Mouse） | *F-Primer* | TTCTTGCGATACACTCTGGTGC |
|  | *R-Primer* | CGGGATTGAATGTTCTTGTCGT |
| *CPT1A*（Human） | *F-Primer* | TCCAGTTGGCTTATCGTGGTG |
|  | *R-Primer* | TCCAGAGTCCGATTGATTTTTGC |
| *Cpt1a*（Mouse） | *F-Primer* | TGGCATCATCACTGGTGTGTT |
|  | *R-Primer* | GTCTAGGGTCCGATTGATCTTTG |
| *MCAD*（Human） | *F-Primer* | ACAGGGGTTCAGACTGCTATT |
|  | *R-Primer* | TCCTCCGTTGGTTATCCACAT |
| *Mcad*（Mouse） | *F-Primer* | AACACAACACTCGAAAGCGG |
|  | *R-Primer* | TTCTGCTGTTCCGTCAACTCA |
| *SCAD*（Human） | *F-Primer* | CGGCAGTTACACACCATCTAC |
|  | *R-Primer* | GCAATGGGAAACAACTCCTTCTC |
| *Scad*（Mouse） | *F-Primer* | GACTGGCGACGGTTACACA |
|  | *R-Primer* | GGCAAAGTCACGGCATGTC |
| *CD36*（Human） | *F-Primer* | GGCTGTGACCGGAACTGTG |
|  | *R-Primer* | AGGTCTCCAACTGGCATTAGAA |
| *Cd36*（Mouse） | *F-Primer* | ATGGGCTGTGATCGGAACTG |
|  | *R-Primer* | TTTGCCACGTCATCTGGGTTT |
| *FATP2*（Human） | *F-Primer* | TTTCCGCCATCTACACAGTCC |
|  | *R-Primer* | CGTAGGTGAGAGTCTCGTCG |
| *Fatp2*（Mouse） | *F-Primer* | CGAGACGAGACGCTCACCTA |
|  | *R-Primer* | ACGAATGTTGTAGTTGAGGCAC |
| *FATP5*（Human） | *F-Primer* | TGAAGGAGCTAGGAGTGGGAA |
|  | *R-Primer* | TGCACCATCTGTAAAGTTGCAG |
| *Fatp5*（Mouse） | *F-Primer* | TCTATGGCCTAAAGTTCAGGCG |
|  | *R-Primer* | CTTGCCGCTCTAAAGCATCC |
| *ACTB*（Human） | *F-Primer* | CATGTACGTTGCTATCCAGGC |
|  | *R-Primer* | CTCCTTAATGTCACGCACGAT |
| *Actb*（Mouse） | *F-Primer* | GTGACGTTGACATCCGTAAAGA |
|  | *R-Primer* | GCCGGACTCATCGTACTCC |

## Reagents and tools table

| Reagent/Resource | SOURCE | IDENTIFIER |
| --- | --- | --- |
| Antibodies | | |
| rabbit anti-TSP50 | Abcam | Cat# ab181993 |
| rabbit anti-SCD1 | Abcam | Cat# ab236868,RRID：AB_2928123 |
| rabbit anti-FASN | Cell Signaling Technology | Cat# 3180, RRID:AB_2100796 |
| rabbit anti-SREBP1 | Abcam | Cat# ab313881 |
| rabbit anti-ACC | Cell Signaling Technology | Cat# 3676, RRID:AB_2219397 |
| rabbit anti-F4/80 | Proteintech | Cat# 28463-1-AP, RRID:AB_2881149 |
| rabbit anti-Ki67 | ZSGB-BIO | Cat# ZA-0502 |
| rabbit anti-AFP | Proteintech | Cat# 14550-1-AP, RRID:AB_2223933 |
| mouse anti-ACTB | Proteintech | Cat# 66009-1-Ig, RRID:AB_2687938 |
| rabbit anti-TUBULIN | Proteintech | Cat# 10094-1-AP, RRID:AB_2210695 |
| Bacterial and virus strains | | |
| BL21 | Beyotime | Cat# D0337 |
| AAV9-mCherry | Justscience | N/A |
| AAV9-mCherry-Tsp50-Flag | Justscience | N/A |
| Chemicals, peptides, and recombinant proteins | | |
| MK-8245 | MedChemExpress | Cat# HY-13070 |
| streptozotocin | Solarbio | Cat# S8050 |
| Tamoxifen | Merk-Sigma | Cat# T5648 |
| Protein A/G Magnetic Beads | MedChemExpress | Cat# HY-K0202 |
| GST Magnetic Beads | MedChemExpress | Cat# HY-K0222 |
| GST-tag Protein Purification Kit | Beyotime | Cat# P2262 |
| Critical commercial assays | | |
| TG Assay Kit | Nanjing Jiancheng Bioengineering Institute | Cat# A110-1-1 |
| TCHO Assay Kit | Nanjing Jiancheng Bioengineering Institute | Cat# A111-1-1 |
| ALT Assay Kit | Nanjing Jiancheng Bioengineering Institute | Cat# C009-2-1 |
| AST Assay Kit | Nanjing Jiancheng Bioengineering Institute | Cat# C010-2-1 |
| Experimental models: Cell lines | | |
| HEK-293T | National Collection of Authenticated Cell Cultures | Cat# GNHu17 |
| HepG2 | National Collection of Authenticated Cell Cultures | Cat# SCSP-510 |
| THLE2 | MeiSen | Cat# CTCC-004-0030 |
| Experimental models: Organisms/strains | | |
| *Tsp50^-/-^* | GemPharmatech | Cat# T012499 |
| Alb-Cre | GemPharmatech | Cat# T017784 |
| *Tsp50^fl/fl^* | GemPharmatech | Cat# T009158 |
| *Tsp50^fl/fl^Alb^Cre^* | This paper | N/A |
| Recombinant DNA | | |
| pcDNA3.0 | This paper | N/A |
| TSP50-FLAG-pcDNA3.0 | This paper | N/A |
| TSP50-H153A-FLAG-pcDNA3.0 | This paper | N/A |
| TSP50-D206A-FLAG-pcDNA3.0 | This paper | N/A |
| TSP50-T310A-FLAG-pcDNA3.0 | This paper | N/A |
| SCD1-MYC- pcDNA3.0 | This paper | N/A |
| pGEX-4T-1-GST | This paper | N/A |
| pGEX-4T-1-GST-TSP50 | This paper | N/A |
| Software and algorithms | | |
| ImageJ Pro Plus 6.0 | National Institue of Health, USA | N/A |
| Zen Vision Care | Zeiss | N/A |
| Prsim 9.0 | GraphPad Software | N/A |
